# Global prevalence of metabolic syndrome in adults with obstructive sleep apnea: a systematic review and meta-analysis

**DOI:** 10.1093/sleep/zsag144

**Published:** 2026-05-22

**Authors:** Suman Pant, Ioannis Kyrou, Alexander Dallaway, Chris Kite, Lukasz Lagojda, Ali Asad, Ashesh Dhungana, Harpal Singh Randeva, Om Prakash Kurmi

**Affiliations:** Discoveries in Life Sciences Research Centre, Coventry University, Coventry, West Midlands, CV1 5FB, United Kingdom; Discoveries in Life Sciences Research Centre, Coventry University, Coventry, West Midlands, CV1 5FB, United Kingdom; Warwickshire Institute for the Study of Diabetes, Endocrinology and Metabolism (WISDEM), University Hospitals Coventry and Warwickshire NHS Trust, Coventry, West Midlands, CV2 2DX, United Kingdom; Institute for Cardiometabolic Medicine, University Hospitals Coventry and Warwickshire NHS Trust, Coventry, West Midlands, CV2 2DX, United Kingdom; Aston Medical School, College of Health and Life Sciences, Aston University, Birmingham, West Midlands, B4 7ET, United Kingdom; College of Health, Psychology and Social Care, University of Derby, Derby, Derbyshire, DE22 1GB, United Kingdom; Warwickshire Institute for the Study of Diabetes, Endocrinology and Metabolism (WISDEM), University Hospitals Coventry and Warwickshire NHS Trust, Coventry, West Midlands, CV2 2DX, United Kingdom; School of Health and Wellbeing, Faculty of Education, Health & Wellbeing, University of Wolverhampton, Wolverhampton, West Midlands, WV1 1LY, United Kingdom; Warwickshire Institute for the Study of Diabetes, Endocrinology and Metabolism (WISDEM), University Hospitals Coventry and Warwickshire NHS Trust, Coventry, West Midlands, CV2 2DX, United Kingdom; School of Health and Wellbeing, Faculty of Education, Health & Wellbeing, University of Wolverhampton, Wolverhampton, West Midlands, WV1 1LY, United Kingdom; Warwickshire Institute for the Study of Diabetes, Endocrinology and Metabolism (WISDEM), University Hospitals Coventry and Warwickshire NHS Trust, Coventry, West Midlands, CV2 2DX, United Kingdom; Sheffield Centre for Health and Related Research (ScHARR), School of Medicine and Population Health, Sheffield, South Yorkshire, S1 4DA, United Kingdom; Department of Respiratory Medicine, University Hospitals Coventry and Warwickshire NHS Trust, Coventry, West Midlands, CV2 2DX, United Kingdom; Department of Pulmonary, Critical Care and Sleep Medicine, National Academy of Medical Sciences, Kathmandu, Bagmati Province, Nepal; Discoveries in Life Sciences Research Centre, Coventry University, Coventry, West Midlands, CV1 5FB, United Kingdom; Warwickshire Institute for the Study of Diabetes, Endocrinology and Metabolism (WISDEM), University Hospitals Coventry and Warwickshire NHS Trust, Coventry, West Midlands, CV2 2DX, United Kingdom; Institute for Cardiometabolic Medicine, University Hospitals Coventry and Warwickshire NHS Trust, Coventry, West Midlands, CV2 2DX, United Kingdom; Warwick Medical School, University of Warwick, Coventry, West Midlands, CV4 7AL, United Kingdom; Department of Pulmonary, Critical Care and Sleep Medicine, National Academy of Medical Sciences, Kathmandu, Bagmati Province, Nepal; Division of Respirology, Department of Medicine, McMaster University, Hamilton, Ontario, Canada; Lincoln Institute for Rural and Coastal Health, University of Lincoln, Brayford Street, Lincoln, Lincolnshire, LN6 7TS, United Kingdom

**Keywords:** obstructive sleep apnea, metabolic syndrome, adults, systematic review, meta-analysis

## Abstract

**Study objectives:**

Metabolic syndrome (MetS) is considered to exhibit increased prevalence among adults with obstructive sleep apnea (OSA), but the reported prevalence estimates among such patients vary. Thus, this systematic review and meta-analysis aimed to investigate the global prevalence of MetS in adults with confirmed OSA.

**Materials and methods:**

Ovid Medline, Embase, CINAHL, and the Cochrane Library databases were searched for all primary studies published in English that used standard polysomnography for OSA diagnosis and reported MetS estimates. At least two reviewers independently screened for eligible studies, extracted data, and graded the risk of bias using the Risk of Bias Assessment Tool for Non-randomised Studies. Three-level random-effects model was applied for the meta-analysis, reporting pooled prevalence estimate with 95% confidence intervals (CIs). Pre-specified subgroup analyses and meta-regression were also performed. Heterogeneity was quantified using *I^2^* and chi-square statistics.

**Results:**

A total of 102 studies were eligible for inclusion (34 013 adults with OSA from 28 countries). The combined MetS prevalence was 55.4% (95% CI: 51.0%, 59.8%). Considerable heterogeneity was noted among the included studies (*I^2^* = 97.8%), whilst the risk of bias ranged from low to high. Subgroup analysis examining the effects of geographic region, study design, MetS definition, and apnea-hypopnea index threshold showed a significant variation in prevalence estimates across most subgroups (*p* < 0.0001). Meta-regression analysis indicated a positive association between mean body mass index (β = 0.0772, t = 4.56, *p* < 0.0001) and MetS prevalence.

**Conclusions:**

MetS has a high prevalence among adults with polysomnography-confirmed OSA, underscoring the need for prompt MetS screening in these patients. Future longitudinal and genetic/mechanistic studies should investigate the factors accounting for this association.

**PROSPERO registration number:**

CRD420251073055.

## Introduction

Obstructive Sleep Apnea (OSA) is an increasingly prevalent chronic sleep-related breathing disorder, typically characterized by repetitive, sudden episodes of partial or complete upper pharyngeal airway closure during the nocturnal sleep cycle, causing apnea, snoring, intermittent hypoxia, sleep fragmentation, and daytime hypersomnolence [1]. Although OSA affects the daily life of nearly a billion adults worldwide [2], an estimated 80-90% of adults with OSA remain undiagnosed [3], indicating that the condition is frequently overlooked and inadequately addressed. The increasing prevalence of obesity and obesity-related metabolic diseases (e.g. type 2 diabetes, hypertension and dyslipidaemia), as well as the increasing ageing of the adult population globally—all established risk factors for OSA [4–6]—have positioned OSA as a pressing global health concern requiring renewed attention.

Metabolic syndrome (MetS; initially termed Syndrome X) represents a clustering of certain interrelated cardio-metabolic diseases, including obesity (particularly central), type 2 diabetes, hypertension and dyslipidaemia [7–9]. Thus, MetS is typically diagnosed based on the presence of at least three of these cardio-metabolic diseases; however, multiple national and international definitions exist, including those by the World Health Organization (WHO) [10], the National Cholesterol Education Program Adult Treatment Panel III (NCEP ATP III) [9, 11], and the International Diabetes Federation (IDF) [12]- each differing in their specific diagnostic criteria, cut-off values, and whether central obesity is a mandatory component. These variations in definition reflect population-specific differences in obesity measures, waist circumference thresholds, and ethnic considerations. OSA and MetS are frequently associated, with the OSA-MetS co-existence being historically referred to as “Syndrome Z” [13] or “two circumferences in the same individual”, [14] suggesting a shared cardio-metabolic substrate. This dyad aggravates the long-term morbidity and mortality of cardiovascular disease [15], and impairs health-related life quality [16, 17]. OSA and MetS are considered to share common biological pathways, overlapping risk factors, and feedback loops, reflecting a complex bidirectional interplay. Indeed, there are data on underlying pathophysiological mechanisms [18–20], the temporal sequence of disease occurrence [21], and the efficacy of interventions [22, 23] which suggest a causative role of OSA in the pathogenesis of MetS, although the predominant/causative direction of the underlying pathogenetic association remains debated.

The presence of MetS in patients with OSA further stratifies risk and modifies treatment needs and responses [16, 24, 25]. Interestingly, although OSA frequency increases with age and is more prevalent among older adults [2], younger adults may also exhibit severe forms of OSA with corresponding metabolic dysfunctions [26]. Overall, men are more prone to OSA, whilst the risk in females significantly increases after menopause [27]. In parallel, MetS and cardiovascular disease are increasing in women, with obesity strongly linked to insulin-resistance related cardio-metabolic risks [27]. Epidemiological studies show that postmenopausal women experience stronger associations between OSA and MetS components than premenopausal women [28, 29], implicating sex and/or menopause as non-modifiable drivers. Lam and colleagues [30] suggested that visceral obesity contributes significantly to the pathophysiology of OSA and to the metabolic derangements associated with the MetS. Conversely, Kumor and colleagues [31] suggested that despite an evident correlation between the apnea-hypopnea index (AHI) and the presence of MetS, no such relationship could be confirmed between transient hypoxia and MetS. Furthermore, the diverse definitions of MetS [32] lead to variations in disease-prevalence reporting, even within individual population subsets [33]. Notably, regardless of any given definition, prevalence estimates increase with age and differ by sex and ethnicity [33].

Despite growing interest in the association between OSA and MetS, the true prevalence of MetS among adults with OSA remains uncertain. Individual studies from India, China, Korea, and Portugal reported highly variable prevalence estimates ranging from 34% to 70% [34–37], influenced by factors such as the MetS definition, OSA severity, sex distribution, and body mass index (BMI). In the context of MetS and OSA, two meta-analyses were published almost a decade ago [38, 39]; however, both concentrated primarily on the risk associations between OSA and MetS rather than on establishing a pooled prevalence estimate. As a result, clinicians and researchers lack an up-to-date, evidence-based estimate of how prevalent MetS is in the global adult population, which can help to support early screening, prevention, and treatment strategies and pathways as part of an integrated sleep-cardio-metabolic care approach. Therefore, the present systematic review and meta-analysis aimed to provide an updated synthesis of evidence on the prevalence estimates of MetS among adults with polysomnography-confirmed OSA and explore potential drivers of heterogeneity in the existing evidence.

## Materials and methods

The study protocol was prospectively registered on PROSPERO (CRD420251073055). Methodological decisions were guided by the Cochrane Handbook [40], and the Preferred Reporting Items for Systematic Reviews and Meta-Analyses (PRISMA) guidelines were followed for reporting standards [41].

### Inclusion criteria

Predefined eligibility criteria were applied based on the Population, Exposure, Comparator, Outcome, Study design (PECOS) framework [42] [i.e. Participants: Adults; Exposure: Obstructive Sleep Apnea; Comparator: Not applicable; Outcome: Prevalence or incidence of MetS; and Study characteristics: observational studies, and baseline (cross-sectional) data of cohort and experimental studies]; detailed in Supplementary Table S1.

### Search strategy and study selection

A predefined electronic search was performed in Medline, Embase, CINAHL and Cochrane Library, from inception to 27^th^ of November 2024, applying a combination of controlled vocabulary and free-text terms related to “obstructive sleep apnea” and “metabolic syndrome”. The search strategy applied for Medline (via Ovid) is presented in Table 1, while the remaining strategies are detailed in Supplementary Tables S2–S4.

**Table 1:** Articles using search terms for Ovid Medline

#	Searches
1.	exp Sleep Apnea, Obstructive
2.	obstructive sleep apn?ea.ti
3.	(sleep* adj3 (apn?ea* or respirat* or breath*)).mp.
4.	hypopn?ea*.ti,ab,tw.
5.	(OSA or SHS or OSAHS or SAHS).tw.
6.	or/1-5
7.	exp Metabolic Syndrome
8.	(“metabolic syndrome” or “Insulin resistance syndrome” or “syndrome x” or “dysmetabolic syndrome” or MetS).mp.
9.	or/7-8
10.	6 and 9

Initially, all retrieved records were imported into EndNote, and duplicates were removed. Two independent reviewers performed the title/abstract and full-text screening using Covidence [43]. At each stage, any uncertainty or disagreement between the two independent reviewers was resolved through consensus and, where required, a third reviewer provided independent arbitration.

To complement the standard search of the aforementioned databases, forward citation chasing was also performed in July 2025 to identify any additional eligible publications. For this citation chasing, eligible studies meeting the inclusion criteria were used to retrieve the lists of cited records using a citation chaser tool [44].

### Data extraction

A standardized data extraction form was developed and reviewed by all authors. Summary datasets were prepared, including key study characteristics, and formatted according to the requirements for statistical analyses.

All eligible studies were assessed to identify potential study clusters and overlapping participants/populations. In instances where the same authors published two or more duplicate articles reporting data from an identical or substantially overlapping population with similar characteristics and outcomes, only the publication providing the most recent, comprehensive, and complete dataset was retained as the primary reference for inclusion in the quantitative synthesis. When relevant information was incomplete, unclear or inaccessible in the identified studies, the corresponding authors were contacted to obtain the missing data or clarification. Where the contacted authors did not respond to these requests, the corresponding studies were excluded from the analyses.

### Assessing risk of bias

The risk of bias for each included eligible study was evaluated by two independent reviewers using the Risk of Bias Assessment Tool for Non-randomized Studies (RoBANS) [45]. This tool includes the following domains: participant selection, confounding variables, measurement of exposure, blinding of outcome assessments, incomplete outcome data, and selective outcome reporting. Each domain is rated for a low, high, or unclear risk of bias. Disagreements were resolved through consensus among the two reviewers.

### Synthesis of results and statistical methods

#### Primary three-level random-effects model

Studies included in this meta-analysis differed in country of origin, study design, MetS diagnostic criteria, AHI threshold used to define OSA, and year of publication. These methodological and clinical differences were expected to introduce significant between-study heterogeneity, supporting the use of a random-effect modelling framework.

A total of 18 clusters, providing 48 prevalence estimates, and 61 studies, each contributing a single estimate, were identified. Clusters were defined based on shared hospital settings or underlying study populations (longitudinal cohorts) reported in the primary studies. Multiple estimates within clusters derived from one or more of the following: (i) analyses conducted by different research teams using the same population or hospital setting; (ii) application of different diagnostic criteria for MetS within a study population; (iii) repeated reporting by the same authors across different years or sub-group-specific estimates (*e.g.* gender) from the same sample. A three-level meta-analytic model [46, 47] was applied to account for this nested data structure while retaining all available estimates.

Within this framework, Level 1 variance represented sampling error across 109 prevalence estimates. Level 2 variance captured variability among the 48 estimates nested within the 18 multi-estimate clusters. Level 3 variance represented variability among independent sources, comprising 18 multiple-estimate clusters and 61 single-estimate studies, treated as clusters of size one. Model fit was evaluated by comparing the three-level model with a conventional two-level model using likelihood-ratio testing and information criteria (AIC and BIC).

Observed prevalence proportions were logit-transformed prior to analysis to normalize their distribution and stabilize variances across studies. Model estimates were back-transformed to prevalence percentages using the inverse-logit transformation for reporting and interpretation. Heterogeneity was quantified at each hierarchical level along with total variance, and variance components were visualized using tree plots generated with the *metafor* package [48]. I-squared statistics (*I^2^*) were interpreted following Cochrane guidelines: 0-40% (not important), 30-60% (moderate), 50-90% (substantial), and 75-100% (considerable) [40]. To further describe between-study variability, 95% prediction intervals were estimated and displayed on a forest plot. Confidence intervals (CIs) reflect the precision of the pooled estimates, whereas prediction intervals indicate the range within which true prevalence is expected across comparable populations in the future.

#### Moderator analyses

Categorical moderators were examined using three-level random-effects subgroup analyses. Countries were grouped according to United Nations (UN) geographical regions based on the *Standard country or area codes for statistical use (M49)* [49]. The details for the different definitions and diagnostic criteria of MetS are presented in Supplementary Table S5.

Continuous moderators were examined using multivariable three-level meta-regression. Age and BMI were mean-centred before inclusion in the model, alongside the percentage of male participants.

For studies reporting mean ± Standard Error of the Mean, mean with CIs, or median with interquartile range, values were converted to mean ± standard deviation (SD) using established methods [50, 51]. When studies reported multiple participant groups with OSA (e.g. severity strata or comorbidity subgroups), group-specific means and SDs were aggregated using sample-size weighting.

Moderator effects were evaluated using omnibus F-tests to assess whether moderators explained variability in prevalence estimates. The QE statistic was used to determine whether significant heterogeneity remained after accounting for moderators.

#### Sensitivity analysis

Sensitivity analyses were conducted to assess the robustness of the pooled estimates and identify influential studies. Baujat plot was used to visualize each study’s contribution to overall heterogeneity and influence on the pooled estimates [52]. Leave-one-out analysis was performed by systematically removing individual studies and recalculating the pooled estimate to determine whether any single study disproportionately affected the results [53].

#### Publication bias

Publication bias was explored descriptively using funnel plots. Formal statistical tests for funnel plot asymmetry (Egger’s regression test) were not performed because the meta-analysis demonstrated extremely high overall heterogeneity. In the presence of marked heterogeneity, funnel plot asymmetry may reflect genuine between-study differences or small-study effects rather than selective publication, rendering asymmetry tests difficult to interpret and potentially misleading [54, 55]. Therefore, a funnel plot was presented for descriptive purposes only and should be interpreted with caution (Supplementary Figure S1).

#### Software

All analysis were conducted in R version 4.4.1 for Windows (R Core Team, Vienna, Austria; www.R-project.org). Three-level meta-analytic models were fitted using the *rma.mv* function from the *metafor* package [48]. The *meta* package [56] was used for visualisation, sensitivity analyses, influence diagnostics, and complementary prevalence estimation using the *metaprop* function with Hartung-Knapp CIs. Clustered analyses were based on inverse-variance weighting, while standard two-level analyses were conducted using the generalized linear mixed model approach. Pooled estimates and CIs were consistent across methods. Analytical syntax was adapted from previously published methodological studies [57, 58].

## Results

### Search results

**Figure 1:**
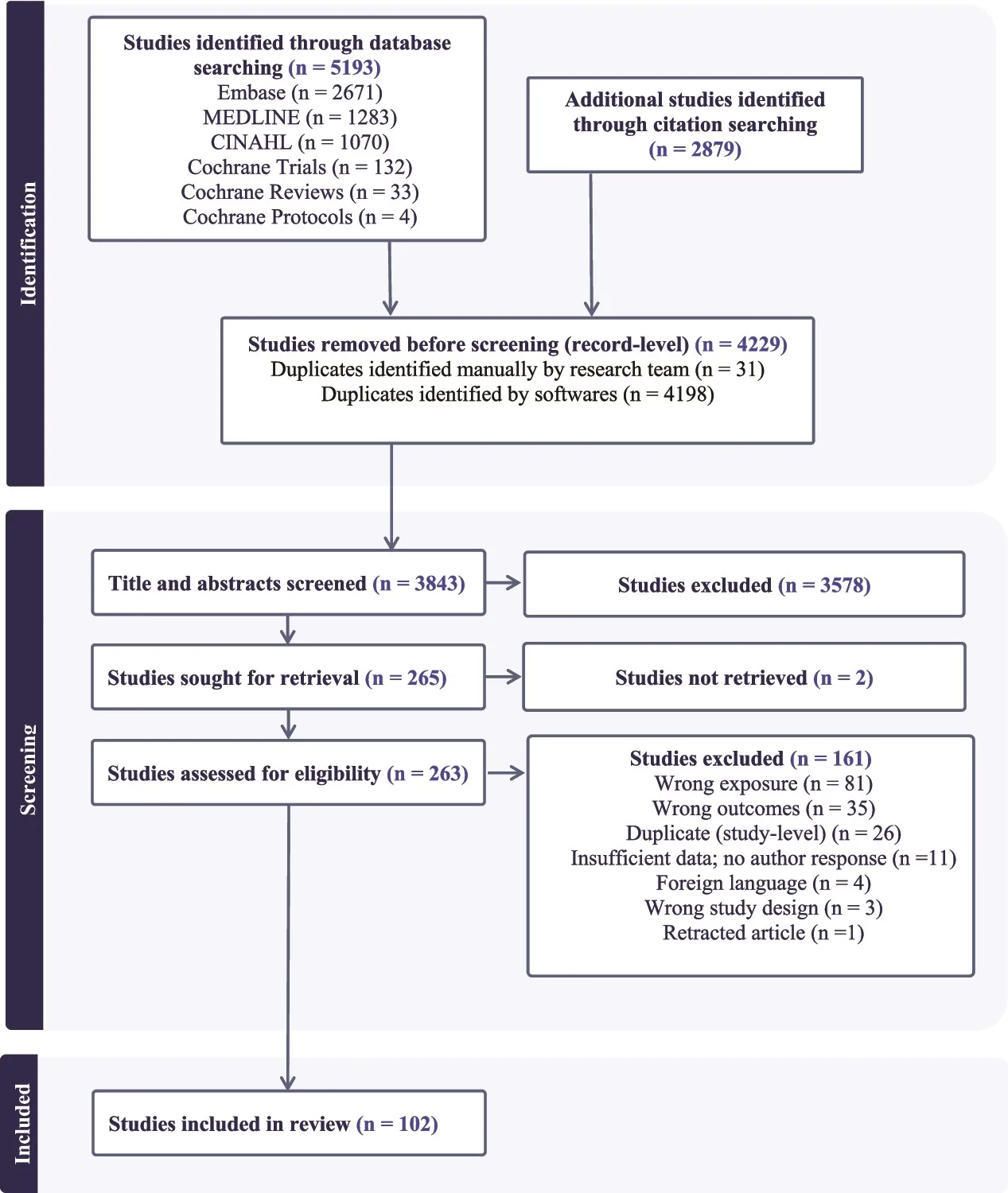
PRISMA flow diagram of the study selection process.

The study selection process is presented in the PRISMA flow diagram of Figure 1. The performed searches retrieved 8072 records, of which 4229 were duplicates. After de-duplication, 3843 unique records were screened. At the title and abstract screening stage, 3578 studies were excluded, resulting in 265 records for full text review. Despite all possible efforts, two full-text records could not be retrieved [59, 60]. The remaining 263 full-text articles were assessed for eligibility, and 161 of these were excluded with reasons, resulting in 102 articles meeting the eligibility criteria and therefore included in this systematic review.

### Characteristics of the included studies

Supplementary Table S6 presents the characteristics of all eligible studies included in the quantitative analysis. A total of 102 studies [20, 21, 30, 31, 34, 36, 61–156] (*k* = 109 effect sizes, with each effect size treated as a separate estimate) comprised 34 013 participants with polysomnography-confirmed OSA, of which 17 274 individuals had MetS. The sample sizes of patients with polysomnography-confirmed OSA in each study ranged from 21 to 3779, with included participants having a mean age ranging from 39.2 (± 7.8) [111] to 67.7 (± 9.0) [147] years, and BMI from 25.1(± 10.3) [116] to 47.4 (± 6.1) [133] kg/m^2^.

The identified eligible articles were published between 2004 and 2025 and represented 12 United Nations geographic regions: Asia (*k* = 58, 53.2%), Europe (*k* = 43, 39.5%), the Americas (4.6%), Africa (1.8%) and Oceania (0.9%). Only 20 studies reported the participants’ ethnicity and race, mostly Caucasian and Han Chinese. A total of 82 cross-sectional studies, 10 case–control studies [73, 74, 77, 80, 84, 119, 131, 132, 136, 137], and baseline data of four randomized controlled trials (RCT) [85, 86, 95, 133], three cohort [21, 36, 108] and three quasi-experimental studies [71, 101, 122] were included in the analysis. Two studies exclusively included female participants with OSA [97, 156], and 14 enrolled males only [64, 84, 85, 93, 98, 103, 115, 119, 121, 124, 127, 129, 144, 151]. Seven studies did not report the participants’ sex [21, 81, 86, 109, 138, 143, 155], while the proportion of male subjects in the remaining studies ranged from 27.5% [133] to 93.6% [139].

To define MetS, most studies (55%) employed NCEP ATP III [9, 11] criteria, whilst the MetS harmonizing criteria [8] were the second most frequently applied criteria/definition (15.6% of the included studies); nine studies did not specify the applied MetS criteria [20, 92, 101, 102, 110, 117, 119, 150, 152]. This review comprised studies involving participants with polysomnography-confirmed OSA, predominantly conducted in hospital settings (89.2%), with the remainder from population-based (8.8%) or community (2.0%) cohorts. AHI thresholds varied across studies, with the majority (78%) applying AHI ≥5 events per hour (hr), followed by 12.8%, 7.4%, and 1.8% for ≥15, ≥10, and ≥ 30 events/hr, respectively.

### Assessment of risk of bias across studies

**Figure 2:**
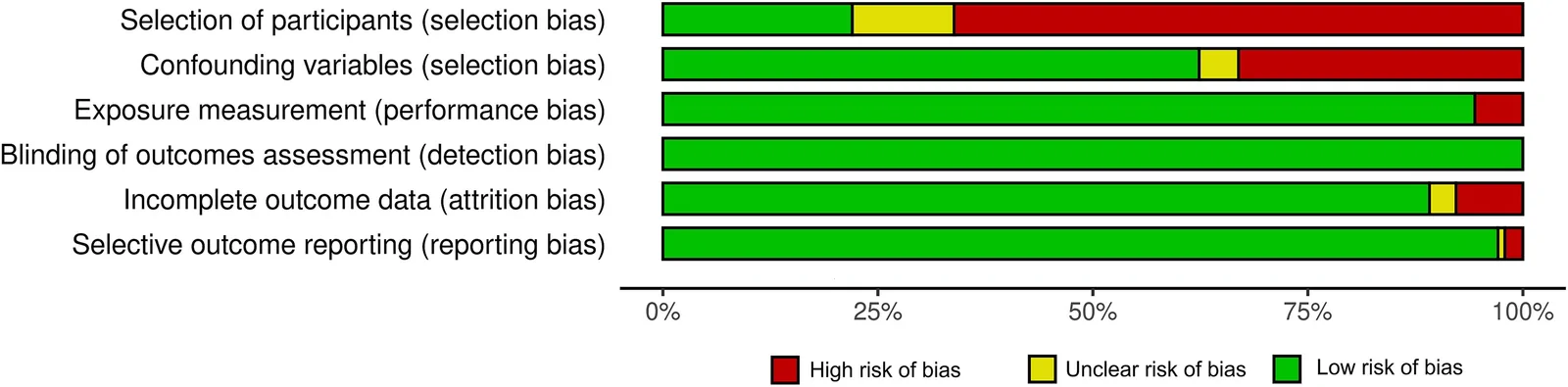
Risk of bias summary for the six domains of the applied RoBANS, presenting the reviewer judgements as percentages for each domain across all 102 included studies.

Figure 2 presents the risk of bias summary for all included studies, as assessed across the six RoBANS domains. A traffic light plot with the risk of bias assessments for each individual eligible study is presented in Supplementary Figure S2.

Across the RoBANS domains, selection bias accounted for the largest proportion (65.7%, 67/102 studies included) of high-risk judgements. This judgement primarily reflected concerns about the representativeness of the included populations. Many studies recruited participants consecutively from a single centre, often from specialized clinics such as diabetes, metabolic, endocrine, or obesity management services. Other studies focused exclusively on clinical populations presenting with sleep-related symptoms undergoing polysomnography, or restricted inclusion to specific subgroups such as individuals with obesity, hypertension, diabetes, or to only males/females. In this domain, 24 (23.5%) studies were rated as low risk, whereas 11 (10.8%) were deemed unclear due to insufficient reporting of study design or recruitment procedures (e.g. whether sampling was random or consecutive).

With respect to confounding, 63 (61.8%) studies demonstrated adequate consideration of, or statistical adjustments for key variables and were therefore rated as low risk. Conversely, 34 (33.3%) studies showed substantial imbalance between comparison groups in factors such as age, sex, anthropometric indices, or biochemical measures. Although these differences were acknowledged and sometimes statistically significant, it was assessed that they were not appropriately addressed during the study design or analysis, resulting in a high-risk judgment. The remaining five studies (4.9%) [65, 86, 121, 148, 152] lacked sufficient information to determine the risk.

For exposure measurement, 97 (95.1%) studies were categorized as low risk, primarily because they used objective polysomnographic recordings. The five studies (4.9%) which had a high risk relied on symptom-based or questionnaire-derived classifications of control participants without physiological confirmation.

In the domain of detection bias, all 102 studies (100%) were considered low risk because the absence of blinding was unlikely to influence the pertinent metabolic outcome measurements, which are typically assessed by laboratory parameters.

Regarding incomplete outcome data, 90 studies (88.2%) were evaluated as low risk, and four (3.9%) [92, 118, 135, 153] as unclear. Eight studies (7.8%) [71, 102, 112, 127, 128, 136, 138, 141] were rated high risk due to substantial attrition (>20%) or missing key variables, such as polysomnography metrics, lipid measures, or waist circumference.

For selective outcome reporting, 99 studies (97%) were assessed as low risk. The studies by Iren et al. [102] and Singh et al. [141] were judged as having a high risk of bias in this domain because critical information of a substantial proportion of participants was missing, and the potential impact of their exclusion on outcome measurement could not be determined. Finally, the study by Vekic and colleagues [149] was judged to have an unclear risk of bias in this domain, because the total number of clinically stable participants who underwent sleep assessment was not reported (only the number of participants diagnosed with OSA was provided).

### Main effects

#### Model fit and global pooled prevalence

**Figure 3:**
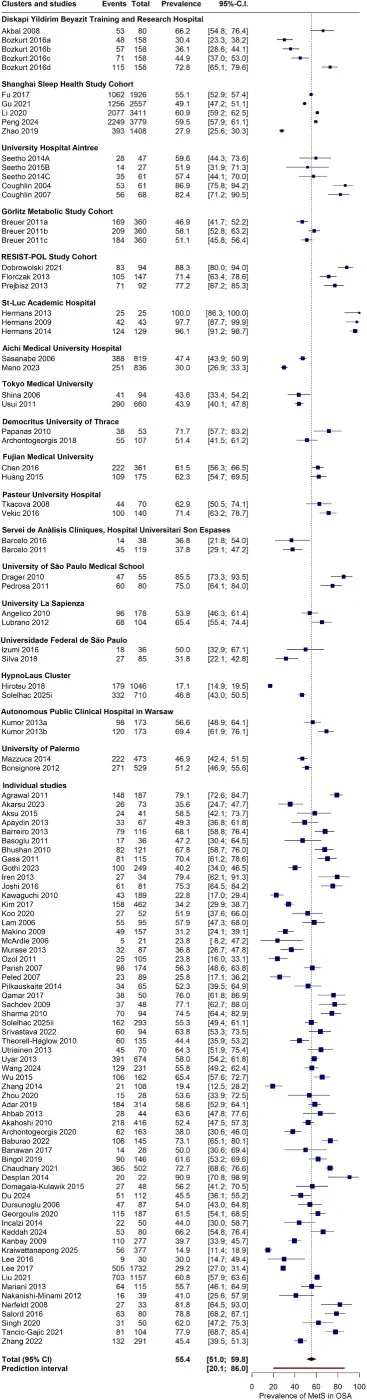
Pooled prevalence of MetS among adults with polysomnography-confirmed OSA. Studies grouped into clusters when multiple estimates originated from the same hospital setting or underlying cohort. “Individual studies” are primary studies that contribute a single prevalence estimate. Bozkurt 2016a–d, represent a single study reporting MetS prevalence using different diagnostic criteria; Breuer 2011a–c, represent multiple estimates derived from different MetS definitions within the same study; Seetho A–C, comprise publications by identical authors with overlapping populations reported across different years; and Solelhac 2025i–ii, represent two cohort datasets from different countries (Switzerland and India) analysed within a single study. “Events” indicate the number of patients with OSA diagnosed with MetS. “Total” denotes the number of patients with OSA included in each study. Horizontal lines represent 95% CI, and the diamond reflects the pooled prevalence estimate with its CI and prediction interval. Abbreviations: CI, confidence interval; MetS, metabolic syndrome; OSA, obstructive sleep apnea.

The overall pooled prevalence of MetS in the three-level meta-analytic model was estimated at 55.4% (95% CI: 51.0%, 59.8%) (Figure 3). Overall heterogeneity was considerable (total *I^2^* = 97.76%). Within-cluster differences (*I^2^*_Level 2_) contributed 39.78% of total variance, whereas between-cluster differences (*I^2^*_Level 3_) constituted the largest proportion (57.98%). The distribution of variance across model levels is illustrated in Supplementary Figure S3 and Table 2.

**Table 2:** Subgroup analysis of prevalence of MetS among adults with polysomnography-confirmed OSA. “*Events”* represent the number of MetS cases, and “*Total”* denotes the number of adults with OSA included in each study. Overall, 102 eligible studies contributed 109 prevalence estimates (*k*). Abbreviations: AHI, apnea-hypopnea index; CI, confidence interval; IDF, International Diabetes Federation; JCCMS, Japanese Committee Of The Criteria For Metabolic Syndrome; MetS: metabolic syndrome; NCEP ATP III: National Cholesterol Education Program Adult Treatment Panel III (Third Report Of The Expert Panel On Detection, Evaluation, And Treatment Of High Blood Cholesterol In Adults); OSA: obstructive sleep apnea; RCT: randomized controlled trial; UN, United Nations; WHO, World Health Organization

Moderator variable	No. of estimates (k)	MetS events	Total OSA	Pooled logit prevalence (95% CI)	Prevalence % (95% CI)	Test of Moderators	Test for Residual Heterogeneity	I ^2^ _Level2_ , %	I ^2^ _Level3_ , %	*Total variance, I* ^2^ , %
**UN geographic regions**						*F*(12, 97) = 3.6922^***^	QE(97) = 2202.8012^***^	47.34	49.75	97.09
Eastern Asia	28	10 607	21 374	-0.2339 (-0.5364; 0.0686)	44.2 (36.9; 51.7)					
Western Asia	17	1342	2665	-0.0839 (-0.4777; 0.3100)	47.9 (38.3; 57.7)					
Southern Europe	16	1376	2531	0.3355 (-0.0606; 0.7316)	58.3 (48.5; 67.5)					
Southern Asia	12	1260	1914	0.7765 (0.3553; 1.1977)	68.5 (58.8; 76.8)					
Western Europe	9	1284	3055	0.7694 (0.0877; 1.4511)	68.3 (52.2; 81.0)					
Eastern Europe	9	675	971	0.8495 (0.2568; 1.4421)	70.0 (56.4; 80.9)					
Northern Europe	9	352	567	0.5463 (-0.0781; 1.1706)	63.3 (48.0; 76.3)					
South America	4	152	256	0.4781 (-0.4273; 1.3836)	61.7 (39.5; 80.0)					
Northern Africa	2	67	108	0.3621 (-0.7127; 1.4369)	59.0 (32.9; 80.8)					
Northern America	1	98	174	0.2542 (-1.1683; 1.6767)	56.3 (23.7; 84.3)					
South-eastern Asia	1	56	377	-1.7461 (-3.1653; -0.3269)	14.9 (4.1; 41.9)					
Australia & New Zealand	1	5	21	-1.1632 (-2.8852; 0.5589)	23.8 (5.3; 63.6)					
**Study designs**						*F*(5, 104) = 3.5872^**^	*QE*(104) = 2594.4810^***^	35.48	62.17	97.65
Cross-sectional	86	15 211	29 585	0.1976 (0.0069; 0.3884)	54.9 (50.2; 59.6)					
Case–control	13	614	1178	0.1540 (-0.3465; 0.6544)	53.8 (41.4; 65.8)					
RCT	4	254	357	1.1756 (0.3863; 1.9650)	76.4 (59.5; 87.7)					
Cohort	3	1040	2665	-0.6795 (-1.4950; 0.1360)	33.6 (18.3; 53.4)					
Quasi-experimental	3	155	228	0.7216 (-0.2426, 1.6858)	67.3 (44.0; 84.4)					
**MetS definitions**						*F*(10, 99) = 2.3173^*^	*QE*(99) = 2486.1712^***^	37.26	60.36	97.62
NCEP ATP III	60	12 899	24 608	0.2812 (0.0602; 0.5022)	57.0 (51.5; 62.3)					
Harmonizing	16	1511	3343	0.3659 (-0.0255; 0.7572)	59.0 (49.4; 68.1)					
IDF	12	755	1277	0.5653 (0.1294; 1.0011)	63.8 (53.2; 73.1)					
National (JCCMS)	6	954	2354	-0.4152 (-1.1079; 0.2775)	39.8 (24.8; 56.9)					
WHO	2	138	304	-0.1485 (-1.0808; 0.7838)	46.3 (25.3; 68.6)					
National (Taiwan)	1	106	162	0.6381 (-0.9362; 2.2123)	65.4 (28.2; 90.1)					
National (China)	1	132	291	-0.1861 (-1.7435; 1.3713)	45.4 (14.9; 79.8)					
Metabolic Vascular System	1	209	360	0.6743 (-0.4858; 1.8343)	66.3 (38.1; 86.2)					
At least one definition	1	115	158	1.4081 (0.2597; 2.5565)	80.4 (56.5; 92.8)					
Not given	9	455	1156	-0.3549 (-0.8788; 0.1689)	41.2 (29.3; 54.2)					
**AHI threshold for OSA diagnosis**						*F*(4, 105) = 2.9808^*^	*QE*(105) = 2688.0646^***^	35.79	61.96	97.75
≥ 5 events/hr	85	14 990	29 670	0.1569 (-0.0395; 0.3533)	53.9 (49.0; 58.7)					
≥ 10 events/hr	8	841	1825	0.0584 (-0.5915; 0.7083)	51.5 (35.6; 67.0)					
≥ 15 events/hr	14	1271	2263	0.6435 (0.2264; 1.0607)	65.6 (55.6; 74.3)					
≥ 30 events/hr	2	172	255	0.6742 (-0.3407; 1.6891)	66.2 (41.6; 84.4)					
**Publication year**						*F*(4, 105) = 2.3731	*QE*(105) =2621.1539^***^	35.30	62.51	97.81
2004-2010	24	1782	3457	0.3985 (0.0768; 0.7202)	59.8 (51.9; 67.3)					
2011-2015	39	3487	6279	0.2246 (-0.0534; 0.5025)	55.6 (48.7; 62.3)					
2016-2020	28	5807	12 593	-0.0007 (-0.3134; 0.3119)	50.0 (42.2; 57.7)					
2021-2025	18	6198	11 684	0.2720 (-0.0872; 0.6313)	56.8 (47.8; 65.3)					

^*^ *p*-value < 0.05.

^**^ *p*-value < 0.01.

^***^ *p*-value < 0.001

The pooled estimate was accompanied by a wide 95% prediction interval (20.1%; 86.0%), indicating that prevalence in future studies conducted in comparable populations may vary substantially.

Model-fit assessment demonstrated that the full three-level model (AIC: 267.4, BIC: 275.4) provided a significantly better fit than the reduced model (AIC: 278.6, BIC: 284.0), as indicated by a significant likelihood-ratio test (LRT = 13.21, *p* = 0.0003).

#### Sensitivity analysis

Influence diagnostics indicated that the majority of studies had minimal impact on model estimates. One study [99] showed elevated influence consistent with summary statistics (Supplementary Figure S4). The Baujat plot revealed seven studies [21, 110, 112, 113, 116, 128, 154] that contributed disproportionately to heterogeneity (Supplementary Figure S5). The reanalysis after removing these eight studies (a new dataset with 101/109 estimates) produced a pooled prevalence of 56.4% (95% CI: 52.6%, 60.3%), consistent with the primary analysis. Heterogeneity remained considerable despite the exclusion of these studies (*I^2^*_Level 2_ = 43.77%, *I^2^*_Level 3_ = 51.89%, total *I^2^* = 95.66%), indicating that the findings were robust to the influence of these studies.

#### Sub-group analysis

As presented in Table 2, moderator analyses using the three-level subgroup models demonstrated significant between-group differences in MetS prevalence across geographic region [F(12, 97) = 3.69, *p* < 0.0001], study designs [F(5, 104) = 3.59, *p* < 0.01], MetS definition/criteria [F(10, 99) = 2.32, *p* < 0.05], and AHI threshold [F(4, 105) = 2.98, *p* < 0.05]. Publication year did not significantly moderate MetS prevalence estimates [F(4, 105) = 2.37, *p* > 0.05].

The highest pooled prevalence was observed across studies conducted in Eastern Europe (70%; 95% CI: 56.4%, 80.9%), closely followed across Southern Asia (68.5%; 95% CI: 58.8%, 76.8%), Western Europe (68.3%; 95% CI: 52.2%, 81.0%), Northern Europe (63.3%, 95% CI: 48.0%, 76.3%), South America (61.7%, 95% CI: 39.5%, 80.0%), and Southern Europe (58.3%, 95% CI: 48.5%, 67.5%). Moderately high prevalence was noticed in Western Asia (47.9%, 95% CI: 38.3%, 57.7%) and Eastern Asia (44.2%, 95% CI: 36.9%, 51.7%). Higher prevalence estimates were present in the baseline data of RCTs (76.4%, 95% CI: 59.5%, 87.7%) and quasi-experimental studies (67.3%, 95% CI: 44.0%, 84.4%) compared to observational designs such as cross-sectional (54.9%, 95% CI: 50.2%, 59.6%) and case–control (53.8%, 95% CI: 41.4%, 65.8%) studies. The lowest MetS prevalence was noted in the baseline assessments of longitudinal cohort studies (33.6%, 95% CI: 18.3%, 53.4%). Regarding applied MetS criteria/definitions, higher pooled prevalence of MetS (63.8%, 95% CI: 53.2%, 73.1%) was noted when studies used the IDF definition, with slightly lower estimates for those that applied the harmonizing (59.0%, 95% CI: 49.4%, 68.1%) or the NCEP ATP III (57.0%, 95% CI: 51.5%, 62.3%) criteria/definition. Studies that applied the WHO definition [10] or the Japanese MetS criteria [157] had lower MetS prevalence estimate (46.3%, 95% CI: 25.3%, 68.6%) and (39.8%, 95% CI: 24.8%, 56.9%), respectively. The included studies used varying AHI cut-off values to define OSA, with thresholds of ≥5, ≥10, ≥15, and ≥ 30 events/hr. Studies were grouped according to the threshold used for OSA case definition rather than disease severity categories. Prevalence estimates increased with higher AHI thresholds. Studies applying a threshold of ≥5 events/hr reported a pooled MetS prevalence of 53.9% (95% CI: 49.0%, 58.7%), while higher thresholds yielded greater estimates, namely 65.6% (95% CI: 55.6%, 74.3%) for ≥15 events/hr and 66.2% (95% CI: 41.6%, 84.4%) for ≥30 events/hr. Studies using a threshold of ≥10 events/hr had a pooled MetS prevalence of 51.5% (95% CI: 35.6%, 67.0%). Finally, regarding publication year, MetS prevalence estimates ranged from 50.0% (95% CI: 42.2%, 57.7%) in studies published between 2016 and 2020 to 59.8% (95% CI: 51.9%, 67.3%) in those published between 2004 and 2010, with estimates of 55.6% (95% CI: 48.7%, 62.3%) and 56.8% (95% CI: 47.8%, 65.3%) for those published during 2011 and 2015, and 2021 and 2025, respectively (Table 2).

**Figure 4:**
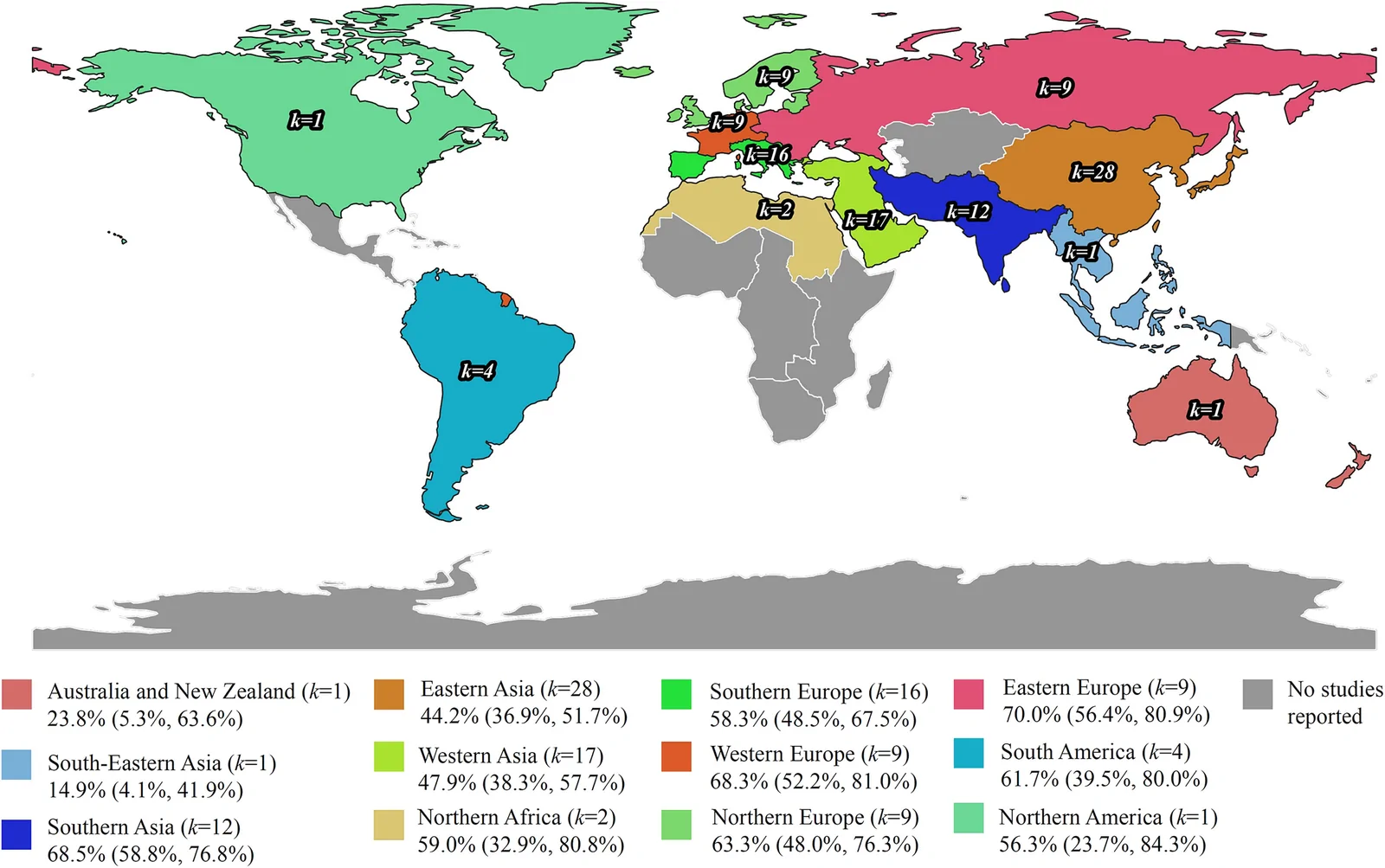
Global distribution of pooled prevalence of MetS among adults with polysomnography-confirmed OSA stratified by UN geographic regions. Regional prevalence estimates were obtained from three-level subgroup meta-analysis and visualized using the QGIS geographic information System [158]. Colour gradients represent pooled prevalence across regions with available data, while grey areas denote regions without data. For each region, the pooled MetS prevalence (%) with corresponding 95% CI is reported, whilst *k* indicates the number of prevalence estimates. Abbreviations: MetS, metabolic syndrome; OSA, obstructive sleep apnea, UN, United Nations.

The initial high overall heterogeneity remained consistent across these subgroups. Residual heterogeneity remained significant within all subgroup models, suggesting that the examined moderators explained only part of the variability in MetS prevalence estimates. Regional prevalence estimates derived from subgroup analysis were visualized on a world map using QGIS [158], as presented in Figure 4.

#### Meta-regression

Meta-regression was performed on 93 studies with complete corresponding moderator data, adjusting for the key covariates. The results showed that, unlike mean age (β = 0.0087, SE = 0.0139, t = 0.63, *p* = 0.53, 95% CI: -0.02, 0.04) and percentage of male sex (β = 0.0060, SE = 0.0042, t = 1.43, *p* = 0.15, 95% CI: -0.002, 0.01), mean BMI was significantly associated with increased effect size (β = 0.0772, SE = 0.0169, t = 4.56, *p* < 0.0001, 95% CI: 0.04, 0.11). Indeed, a 5-unit increase in BMI was associated with 41% higher odds of MetS among adults with OSA (OR: 1.41, 95% CI: 1.21, 1.64), indicating that studies with higher mean BMI reported greater prevalence of MetS. The test for residual heterogeneity was statistically significant (Q_E_ (93) = 1704.7807, *p* < 0.0001), indicating the presence of additional unmeasured moderators. The overall test of these moderators was statistically significant [F (3, 93) = 6.98, *p* < 0.001], indicating that these variables collectively explained a significant proportion of between-study heterogeneity. The bubble plot for the meta-regression examining the effect of mean BMI on the prevalence of MetS in OSA is presented in Supplementary Figure S6.

## Discussion

To the best of our knowledge, this is the first systematic review and meta-analysis investigating specifically the prevalence of MetS among adults with polysomnography-confirmed OSA, including a large number of studies (N = 102) and a pooled number of OSA cases (N = 34 013). It is noteworthy that, based on the applied eligibility criteria, data from studies with confirmed OSA diagnosis were included, relying on peer-reviewed publications/studies with “gold-standard” Level 1 sleep examinations, whilst all established MetS definitions were included. Accordingly, this meta-analysis showed a markedly high (55.4%) global prevalence of MetS among adults with polysomnography-confirmed OSA. This is the most up-to-date and robust estimate of MetS in such patients, generated by pooling data from observational and experimental studies conducted globally (28 countries across 12 UN geographic regions) and employing three-level random-effects models, while also accounting for potential confounding factors. As expected high heterogeneity was noted, explained, at least in part, by the inclusion of more studies [159]. Sensitivity analyses were also performed, which were in agreement with the primary findings.

The combined prevalence estimate of MetS (for the geographic regions with more than one eligible estimate/study; Table 2) from among adults with polysomnography-confirmed OSA showed geographic variability, ranging from 44.2% in Eastern Asia to 70% in Eastern Europe. A large meta-analysis of global data from the general adult population (28 million individuals) reported a global prevalence of MetS between 12.5% and 31.4% according to the definition considered, which also showed geographic variability with significantly higher prevalence in the Eastern Mediterranean Region and the Americas [160]. Notably, this high global prevalence of MetS in the general adult population is lower than the present MetS prevalence estimates among adults with OSA, suggesting that MetS prevalence is even higher in this patient population. The noted geographic variability can be attributed, at least in part, to the complex interplay of inherited body-composition phenotypes, ancestry-specific metabolic genetics, regionally evolving dietary patterns, variable physical activity levels, and differences in clinical diagnosis pathways. For example, genome-wide evidence shows that both overall adiposity phenotypes and body-fat distribution phenotypes are highly polygenic (e.g. numerous genetic loci associated with increased/decreased BMI and waist-to-hip ratio have been identified in predominantly European cohorts [161, 162]). Moreover, South Asian populations (e.g. populations in Indian and other South Asian countries) exhibit high visceral adiposity and insulin resistance at lower BMI, reflecting differences in the risk profile associated with body adiposity distribution/composition [163]. Additionally, the *FTO* variant (Fat Mass and Obesity-associated gene) is associated with increased waist circumference and interacts strongly with physical activity, amplifying susceptibility to central adiposity phenotypes in sedentary individuals [164]. Substantial regional differences also exist in lifestyle exposures which directly influence the cardio-metabolic risk, with existing data suggesting increased levels of insufficient physical activity in Europe and the Americas [165]. Furthermore, dietary factors similarly differ across regions/countries (e.g. South Asian populations increasingly adopt unhealthy diets with refined carbohydrates, sugar-rich beverages, and trans-fatty acids, whilst European populations also tend to follow such dietary habits) [166]. These lifestyle factors are not consistently/adequately reported in studies on the prevalence of MetS in adults with OSA and should be taken into account in the design of future studies in this field, particularly in relation to the noted variability across different regions/countries.

Most of the identified eligible studies originated from Europe and Asia, indicating a greater research focus on cardio-metabolic consequences of OSA in these regions. In contrast, studies from the Americas and Oceania were scarce, resulting in an uneven geographic distribution of the available evidence. This imbalance may influence the pooled estimate prevalence estimates, as regional differences in genetic background, lifestyle factors, obesity prevalence, and healthcare access could affect the coexistence of OSA and MetS. Future epidemiological studies from under-represented regions are needed to improve the global representativeness and external validity of the current evidence base.

Methodological factors may further influence the variability of the reported prevalence of MetS in adults with OSA. In the present dataset, many South Asian and European studies were conducted in cardio-metabolic, or specialist sleep/respiratory centres, increasing the likelihood of recruiting individuals with both OSA and MetS, and thereby potentially introducing a Berkson’s type of selection bias [167]. Conversely, in most studies from East Asia, OSA is often diagnosed in Ear, Nose, and Throat or neurology settings among patients without obesity [93, 109, 111–113, 128], reducing the likelihood of concurrent MetS diagnosis/screening [168]. Moreover, despite the widespread use of the NCEP ATP III MetS criteria across the included studies, the noted regional differences in MetS prevalence is further shaped by the variable genetic and lifestyle background, rather than by MetS diagnostic inconsistencies alone.

The present findings show a higher average MetS prevalence in experimental and clinic-based observational studies, whereas it was lower in population-based cohorts. This may not be random, but rather consistent with methodological aspects, largely attributable to selection bias and the clinical context of study participants in the former study designs [85, 86, 95, 133]. This has also been noted in previous meta-analyses in other fields, such as for tuberculosis among people using drugs [169] or for cardiac abnormalities in COVID-19 [170], which have also reported differences between hospital-based compared with population-based cohort estimates; none, however, captured the full spectrum from RCT to broader cohort designs. The gradient in MetS prevalence among adults with OSA observed in the present analysis (i.e. experimental > quasi-experimental > cross-sectional/case–control > cohort studies) suggests that selection/recruitment bias linked to the study design may not merely be a source of heterogeneity, but also a potential determinant of the reported comorbidity burden. For example, the comparatively higher prevalence of MetS among baseline participants with OSA in RCTs may reflect a more severe comorbidity profile of those typically recruited into Continuous Positive Airway Pressure (CPAP) trials and studies based in specialist sleep clinics [85, 86, 95, 133]. This design-associated heterogeneity in the prevalence of comorbid MetS indicates that such factors should be considered when designing future studies in this field.

Notably, the MetS definition emerged as an important moderator of MetS prevalence among adults with OSA in the present analysis. Among the commonly used MetS definitions/criteria, the IDF criteria yielded higher MetS prevalence (63.8%), which appears to be consistent with including central obesity as a prerequisite for MetS diagnosis and applying ethnicity-specific waist-circumference thresholds. Indeed, both these factors can increase the case detection in populations with a high burden of visceral adiposity, such as those with OSA [171, 172]. In comparison, the harmonized and the NCEP ATPIII MetS definitions produced similar, slightly lower estimates (59.0% and 57.0%, respectively), reflecting their more flexible criteria regarding central obesity, which in these definitions is not a prerequisite for MetS diagnosis [8, 9, 11]. The lowest prevalence (39.8%) was observed when applying the Japanese criteria/definition, which, as expected, was used exclusively in studies conducted in Japan. The JCCMS criteria are applied to Japanese patients/populations with distinct adiposity profiles, and, thus, impose stringent waist-circumference thresholds and require central obesity as an essential condition. Subgroup analyses demonstrated statistically significant MetS prevalence estimates across all definitions (all *p* < 0.0001).

The present analysis also revealed a dose–response trend between the AHI threshold used to diagnose OSA and the pooled MetS prevalence estimates. Indeed, the higher AHI cut-offs for OSA diagnosis, typically reflecting greater OSA severity, were associated with higher MetS prevalence. This trend aligns with established evidence linking OSA severity to visceral adiposity, sympathetic activation, oxidative stress, and insulin resistance [173], which collectively increase the metabolic burden in the more severe phenotype. The slightly higher MetS prevalence observed in the AHI ≥5 events/hr group (53.9%) compared with the one for the AHI ≥10 events/hr group (51.5%), although seemingly counterintuitive, it is likely to reflect, at least in part, differences in study populations or unmeasured confounding in the included studies. However, the overall trend remains, since when the applied AHI threshold for the diagnosis of OSA increases to ≥15 and ≥ 30 events/hr the comorbidity burden of MetS also increases markedly (MetS prevalence of 65.6% and 66.2%, respectively). The similar MetS prevalence estimates for the higher AHI thresholds suggest a potential plateau effect, which can be attributed, at least in part, to two factors. First, key underlying mechanisms linking OSA to MetS (e.g. intermittent hypoxia, sympathetic activation, and sleep fragmentation) are active at moderate severity, with limited additional impact for the diagnosis of MetS at higher AHI levels. Second, at least a minimum degree of obesity (particularly central), which is a key risk factor for both OSA and MetS diagnosis, is present across all OSA severities, possibly confounding any severity-related differences in MetS prevalence, particularly at the higher AHI thresholds. Overall, these findings reinforce the notion that such higher AHI thresholds, beyond their diagnostic role, serve as an indicator of underlying cardio-metabolic stress. They also complement longitudinal evidence demonstrating that more severe OSA is associated with a greater risk of incident metabolic abnormalities [21].

In contrast to other categorical moderators, the publication year of the included eligible studies did not significantly influence the pooled prevalence of MetS. Although minor variations in point estimates were observed across the different publication periods, the prevalence estimates were broadly similar, with substantial overlap in their CIs. This suggests that the burden of MetS among adults with confirmed OSA has remained relatively consistent over time.

In the present meta-regression, a 5-unit increase in mean BMI was associated with 41% greater odds of MetS. This indicates that adiposity is a significant modifiable risk factor linking OSA and MetS, and is consistent with the extensive relevant pathophysiological evidence [173, 174]. By contrast, neither the mean age (*p* = 0.53) nor the percentage of male participants (*p* = 0.15) significantly moderated the noted MetS estimate. This suggests that the metabolic vulnerability associated with confirmed OSA is broadly consistent across the included adult age range (39–68 years) and does not vary substantially according to sex distribution within the identifiable eligible study samples.

Although the included covariates demonstrated a significant joint effect (*p* < 0.0001), the performed meta-regression left substantial residual heterogeneity unexplained (QE (93) = 1704.78, *p* < 0.0001). This indicates that while BMI is a key determinant, between-study variation is largely driven by a combination of other methodological and clinical factors (e.g. the diagnostic criteria for MetS, geographic region, and study design).

### Strength and limitations

#### Evidence perspective

This systematic review and meta-analysis synthesizes evidence from a large, pooled sample size reporting the prevalence of MetS in adults with confirmed OSA. Notably, the OSA diagnosis across all included studies was based on standard polysomnography, ensuring accurate case selection and minimising misclassification with other sleep-disordered breathing phenotypes. The inclusion of studies from diverse geographic regions further enhances the external validity and generalisability of the findings.

A further strength is the inclusion of all established MetS definitions, allowing this review to also explore how MetS prevalence estimates vary by diagnostic definition/criteria. Furthermore, subgroup analyses and meta-regression of covariates enabled a structured examination of potential confounders and effect modifiers in the relationship between OSA and MetS.

However, certain limitations must be acknowledged. Substantial residual heterogeneity persisted despite meta-regression and subgroup analyses, suggesting that important covariates were unmeasured or inconsistently reported across the included eligible primary studies. In particular, ethnicity-related data [175, 176], lifestyle behaviours such as smoking [177, 178] and physical activity [179], menopausal status [179, 180], and other cardio-metabolic determinants were not reported consistently by the identified eligible studies, thus limiting the ability to fully adjust for these known MetS risk factors. Moreover, the predominance of observational study designs limits causal inference, whilst variability in MetS diagnostic criteria may have introduced classification inconsistencies at the study level. Finally, some geographic regions, particularly low-and middle-income countries/regions, remain under-represented, which may influence regional prevalence estimates.

#### Review perspective

This review was designed and conducted by a multidisciplinary team with expertise in sleep medicine, endocrinology, and systematic review/meta-analysis methodology, supporting strong conceptual, clinical and methodological rigour. The applied methodology facilitated a transparent, reproducible, and systematic workflow across database searching (an extensive search strategy of the most relevant databases, supplemented by citation chasing), screening, data extraction, and analysis processes.

The risk of bias was assessed independently by two researchers using a well-validated six-domain tool, enabling a comprehensive appraisal of selection, measurement, and analytical biases across studies. This structural assessment not only strengthens the internal validity but also highlights specific methodological limitations in the existing literature, providing guidance for future high-quality research.

Methodologically, the adoption of three-level random-effects modelling, a reproducible statistical procedure in R, further strengthened the robustness of the present findings. Of note, where studies provided sufficient detail, outcomes and characteristics were organized into clusters, allowing appropriate inclusion of these data and improving interpretability. Furthermore, examining multiple potential moderators enabled a detailed exploration of sources of heterogeneity and provided clinically informative insights. A limitation which relates to the statistical software used was that, although the primary analyses were conducted using the *rma.mv* function in the *metafor* package [48], visualisation and diagnostic tools are not fully integrated within this package, necessitating the use of functions from the *meta* package [56].

Finally, although every effort was made to ensure comprehensive coverage, the possibility of missing studies not registered in the searched databases or non-English studies cannot be entirely excluded.

## Conclusion

The findings of the present systematic review and meta-analysis indicate that nearly six out of 10 adults with a polysomnography-confirmed diagnosis of OSA have MetS. This is both clinically consequential and substantially higher than the estimate of MetS in general adult populations, and, thus, highlights the importance of systematically and promptly screening for MetS in adults with OSA. This high MetS burden among these patients is not attributable to a single factor, but likely arises from an interplay of various factors, including adiposity, cardio-metabolic risk clustering, and demographic variation. While meta-regression highlights BMI as a significant predictor of MetS in patients with OSA, substantial unexplained heterogeneity suggests that additional biological and/or contextual factors play a significant role. These findings warrant more rigorous phenotyping and well-designed large-scale studies to improve OSA-MetS research. Future research should further investigate underlying biological mechanisms, integrating standardized cardio-metabolic assessments, longitudinal designs, and population-level datasets with detailed genetic and biomarker information. Indeed, by highlighting the markedly high prevalence of MetS among adults with OSA, this meta-analysis provides a strong foundation and compelling rationale for leveraging large-scale cohorts to explore causal mechanisms and refine risk stratification in this patient population.

## Supplementary Material

Supplementary_material_zsag144

## Data Availability

The data supporting the findings of this review are available from the corresponding authors upon reasonable request.
