# Supplementary material for: Global prevalence of metabolic syndrome in adults with obstructive sleep apnea: a systematic review and meta-analysis
Source: Sleep. 2026 May 22;49(9):zsag144. doi: 10.1093/sleep/zsag144 (PMC13553307; doi:10.1093/sleep/zsag144)
Supplement: Supplementary file 1 [file supplementary_material_zsag144.zip › OSA and MetS SR - Supplementary File.docx]

**Global prevalence of metabolic syndrome in adults with obstructive sleep apnoea: A systematic review and meta-analysis**

**Supplementary Information**

**Authors and affiliation(s):**

Suman Pant^1^, Ioannis Kyrou^1,2,3,4,5^, Alexander Dallaway^2,6^, Chris Kite^2,6^, Lukasz Lagojda^2,7^, Ali Asad^8^, Ashesh Dhungana^9^, Harpal Singh Randeva^1,2,3,10,^ *, Om Prakash Kurmi^9,11,12,^ *

1. Discoveries in Life Sciences Research Centre, Coventry University, Coventry, CV1 5FB, United Kingdom
2. Warwickshire Institute for the Study of Diabetes, Endocrinology and Metabolism (WISDEM), University Hospitals Coventry and Warwickshire NHS Trust, Coventry, CV2 2DX, United Kingdom
3. Institute for Cardiometabolic Medicine, University Hospitals Coventry and Warwickshire NHS Trust, Coventry, CV2 2DX, United Kingdom
4. Aston Medical School, College of Health and Life Sciences, Aston University, Birmingham, B4 7ET, United Kingdom
5. College of Health, Psychology and Social Care, University of Derby, Derby, DE22 1GB, United Kingdom
6. School of Health and Wellbeing, Faculty of Education, Health & Wellbeing, University of Wolverhampton, Wolverhampton, WV1 1LY, United Kingdom
7. Sheffield Centre for Health and Related Research (SCHARR), School of Medicine and Population Health, Sheffield, S1 4DA, United Kingdom
8. Department of Respiratory Medicine, University Hospitals Coventry and Warwickshire NHS Trust, Coventry, CV2 2DX, United Kingdom
9. Department of Pulmonary, Critical Care and Sleep Medicine, National Academy of Medical Sciences, Kathmandu, Nepal
10. Warwick Medical School, University of Warwick, Coventry, CV4 7AL, United Kingdom
11. Division of Respirology, Department of Medicine, McMaster University, Hamilton, Canada
12. Lincoln Institute for Rural and Coastal Health, University of Lincoln, Brayford Street, Lincoln, LN6 7TS, United Kingdom

***Joint Senior Author**

**Corresponding authors:**

1. Suman Pant, Discoveries in Life Sciences Research Centre, Coventry University, Coventry, CV1 5FB, United Kingdom, [pants2@uni.coventry.ac.uk](mailto:pants2@uni.coventry.ac.uk), +4402477656565
2. Om Prakash Kurmi, Lincoln Institute for Rural and Coastal Health, University of Lincoln, Brayford Street, Lincoln, LN6 7TS, United Kingdom, [okurmi@lincoln.ac.uk](mailto:okurmi@lincoln.ac.uk)

**Supplementary Information**

**Supplementary Table S1.** Eligibility criteria for the present systematic review following the Population, Exposure, Comparator, Outcome, Study design (PECOS) framework.

**Supplementary Table S2.** Search terms/strategy for EMBASE via OVID.

**Supplementary Table S3.** Search terms/strategy for Cochrane Library.

**Supplementary Table S4.** Search terms/strategy for CINAHL via EBSCO*host*.

**Supplementary Table S5.** Definitions and diagnostic criteria applied for the diagnosis of metabolic syndrome in adults.

**Supplementary Table S6.** Key pertinent study characteristics.

**Supplementary Figure S1.** Funnel plot.

**Supplementary Figure S2.** Traffic light plot presenting the assessments for the six domains of the applied Risk of Bias Assessment Tool for Non-randomised Studies (RoBANS) for each of the included studies.

**Supplementary Figure S3.** Tree-plot depicting heterogeneity decomposition across three meta-analysis levels of the applied model.

**Supplementary Figure S4.** Sensitivity analysis plot.

**Supplementary Figure S5.** Baujat plot.

**Supplementary Figure S6.** Meta-regression of BMI and prevalence of MetS in patients with OSA.

**Supplementary Table S1**. Eligibility criteria for the present systematic review following the Population, Exposure, Comparator, Outcome, Study design (PECOS) framework.

**Population**

| **Inclusion** | **Exclusion** |
| --- | --- |
| Adults * (18 years and older) | Paediatric participants  Adolescents  Pregnant participants  Lactating mothers |
| *Note: As an a *priori* inclusion criteria, studies involving mixed age populations (e.g., pediatric and/or adolescent participants) were considered eligible only if data for the relevant adult population could be extracted separately. If the required data were not provided in the published manuscript, we contacted the study authors for clarification and included the study only if the necessary data could be obtained. During the study selection, no studies with mixed age populations fulfilled this criterion; consequently, all studies in the final review comprised exclusively adult participants. | |

**Exposure**

| **Inclusion** | **Exclusion** |
| --- | --- |
| Individuals diagnosed** with Obstructive Sleep Apnoea (OSA).  All severity levels (mild, moderate or severe based on Apnoea-Hypopnea Index)  If Sleep Disordered Breathing (SDB) was reported and further sub-categorized with at least one category reporting OSA based on point 1. | Studies relying solely on questionnaire, pulse oximetry, simplified poly-graph without airflow of effort measurements, or cardiorespiratory monitoring.  Individuals diagnosed broadly with SDB without specific differentiation of OSA.  Studies that report SDB, but do not provide separate data for OSA patients. |
| **Note: Patients diagnosed with OSA by one or more of the following methods: full-night polysomnography (Level I Gold Standard) **OR** ICD9 or 10 codes for classification with documented evidence of polysomnography **OR** medical records indicating that OSA diagnosis was made based on polysomnography findings | |

**Outcome**

| **Inclusion** | **Exclusion** |
| --- | --- |
| Prevalence and incidence of metabolic syndrome | Not reporting outcome-related data |

**Study Characteristics**

| **Inclusion** | **Exclusion** |
| --- | --- |
| Cross-sectional studies  Cohort studies  Case-control studies  Randomized Controlled trials  Quasi-randomized trials  For intervention studies, baseline data were used, as these reflected the initial burden of disease in the study population and avoided mixing incident and prevalent cases. This approach also ensured comparability across studies, given variations in follow-up duration, and reduced potential bias arising from loss to follow-up or intervention effects. | Qualitative studies  Studies lacking original data (i.e. case reports, reviews, editorials, opinions, protocols, conferences, news, systematic reviews, and meta-analyses |

**Others**

| **Inclusion** | **Exclusion** |
| --- | --- |
| Peer-reviewed  English language only  Full-text available  In cases where multiple articles by the same authors report on overlapping populations with similar characteristics and outcomes, only most recent or comprehensive publication were included. | Other language except English  Conference abstracts, academic dissertations, and other grey literature  Animal and *in-vitro* research  Clinical conditions with presentations similar to OSA (e.g., central apnoea) and/or MetS (e.g., Gaucher disease, glucose Galactose malabsorption, Adrenoleukodystrophy, Menkes syndrome, Wilson’s disease, etc.) |
| For incomplete data or information, study authors were contacted and included in case complete information/ data were retrieved. | |

**Supplementary Table S2.** Search terms/strategy for EMBASE via OVID.

| **#** | **Searches** |
| --- | --- |
| 1. | exp sleep apnea syndrome |
| 2. | obstructive sleep apn?ea.ti |
| 3. | (sleep* adj3 (apn?ea* or respirat* or breath*)). ti,ab,tw. |
| 4. | hypopn?ea*.ti,ab,tw. |
| 5. | (OSA or SHS or OSAHS or SAHS).tw. |
| 6. | or/1-5 |
| 7. | exp metabolic syndrome X |
| 8. | ("metabolic syndrome" or "Insulin resistance syndrome" or "syndrome x" or "dysmetabolic syndrome" or MetS).mp. |
| 9. | or/7-8 |
| 10. | 6 and 9 |

**Supplementary Table S3.** Search terms/strategy for Cochrane Library.

| **#** | **Searches** |
| --- | --- |
| **1.** | MeSH descriptor: [Sleep Apnea Syndromes] explode all trees |
| **2.** | (obstructive sleep apn?ea):ti,ab,kw |
| **3.** | sleep* near/3 (apn?ea* or respirat* or breath*) |
| **4.** | (hypopn?ea*):ti,ab,kw |
| **5.** | (OSA or SHS or OSAHS or SAHS) |
| **6.** | {OR #1-#5} |
| **7.** | MeSH descriptor: [Metabolic Syndrome] explode all trees 3640 |
| **8.** | "metabolic syndrome" or "Insulin resistance syndrome" or "syndrome x" or "dysmetabolic syndrome" or MetS |
| **9.** | #7 or #8 |
| **10.** | #6 and #9 |

**Supplementary Table S4.** Search terms/strategy for CINAHL via EBSCO*host*.

|  | **Searches** |
| --- | --- |
| S1 | (MH "Sleep Apnea, Obstructive") |
| S2 | TI obstructive sleep apn#ea OR AB obstructive sleep apn#ea |
| S3 | sleep* N3 (apn#ea* or respirat* or breath*) |
| S4 | TI hypopn#ea* OR AB hypopn#ea* |
| S5 | TX (OSA or SHS or OSAHS or SAHS) |
| S6 | S1 or S2 or S3 or S4 or S5 |
| S7 | (MH "Metabolic Syndrome X") |
| S8 | "metabolic syndrome" or "insulin resistance syndrome" or "syndrome x" or "dysmetabolic syndrome" or MetS |
| S9 | S7 or S8 |
| S10 | S6 and S9 |

**Supplementary Table S5.** Definitions and diagnostic criteria applied for the diagnosis of metabolic syndrome in adults.

| **CRITERIA** | **WHO 1998 [1]** | **NCEP ATP III 2001 [2]** | **AHA/NHLBI 2005 (often referred to as updated NCEP ATP III) [3]** | **IDF 2005 [4]** | **JCCMS 2005 [5]** | **JIS 2009 (Harmonizing criteria by the IDF & the AHA/NHLBI) [6]** |  |
| --- | --- | --- | --- | --- | --- | --- | --- |
| **Prerequisite for diagnosis** | Insulin resistance (IFG, IGT, T2DM or other evidence) | None | None | Central obesity defined based on ethnic-related WC cut-off points:  - European ancestry: WC ≥94 cm (male), ≥80 cm (female)  - South Asians, Chinese and Japanese: WC ≥90 cm (male), ≥80 cm (female)  - South and Central Americans: Use South Asian cut-offs until more specific data are available  - Sub- Saharan Africans & East Mediterranean and Middle East (Arab): Use of European data until more specific data are available | Visceral fat accumulation (WC) | None |  |
| **Diagnostic criteria** | Insulin resistance or T2DM, and at least two of the five criteria below: | At least three of the following five criteria: | At least three of the following five criteria: | Central obesity, and at least two of the following four criteria: | WC and at least two of the following: | At least three of the following five criteria: |  |
| **Obesity/WC related indices (e.g. WC or WHR)** | WHR >0.90 cm in males, >0.85 cm in females and/or BMI >30 kg/m² | WC ≥102 cm in males, ≥88 cm in females | WC ≥102 cm in males, ≥88 cm in females | Central obesity as detailed above | WC ≥85 cm in males, ≥90 cm in females, corresponding to visceral fat area ≥100 cm^2^ on CT scan | WC over the population-and country-specific cut-offs recommended by the IDF for non-Europeans and either the IDF or AHA/NHLBI cut-offs used for Europids until more data are available |  |
| **Dyslipidaemia: TG** | ≥150 mg/dl (1.7 mmol/L) | ≥150 mg/dl (1.7 mmol/L) | ≥150 mg/dl (1.7 mmol/L) or receiving pharmacological treatment | TG ≥150 mg/dl (1.7 mmol/L) or receiving pharmacological treatment | ≥150 mg/dl (1.7 mmol/L) | ≥150 mg/dl (1.7 mmol/L) or receiving pharmacological treatment |  |
| **Dyslipidaemia: HDL-C** | <35 mg/dl (0.9 mmol/L) in males, <39 mg/dl (1.0 mmol/L) in females | <40 mg/dl (1.04 mmol/L) in males, <50 mg/dl (1.33 mmol/L) in females or receiving treatment | <40 mg/dl (1.04 mmol/L) in males, <50 mg/dl (1.33 mmol/L) in females, or receiving treatment | <40 mg/dl (1.04 mmol/L) in males, <50 mg/dl (1.33 mmol/L) in females, or receiving treatment | <40mg/dl (1.04 mmol/L) (both sexes) | <40 mg/dl (1.04 mmol/L) in males, <50 mg/dl (1.33 mmol/L) in females, or receiving treatment |  |
| **BP** | ≥140 mmHg systolic or ≥90 mmHg diastolic or receiving antihypertensive medication | ≥130 mmHg systolic or ≥85 mmHg diastolic or receiving antihypertensive medication | ≥130 mmHg systolic or ≥85 mmHg diastolic or receiving antihypertensive medication | ≥130 mmHg systolic or ≥85 mmHg diastolic or receiving antihypertensive medication | ≥130 mmHg systolic and/or ≥85 mmHg diastolic or receiving antihypertensive medication | ≥130 mmHg systolic or ≥85 mmHg diastolic or receiving antihypertensive medication |  |
| **Fasting Glucose** | Insulin resistance already required | ≥110 mg/dl (6.1 mmol/L) (includes diabetic individuals) | ≥100 mg/dl (5.6 mmol/L) or on pharmacological treatment | ≥100 mg/dl (5.6 mmol/L) or receiving treatment | ≥110 mg/dl (6.1 mmol/L) or receiving pharmacological treatment | ≥100 mg/dl (5.6 mmol/L) or previously diagnosed T2DM |  |
| **Other required criteria** | Urinary albumin excretion of ≥20 mcg/minute or albumin-to-creatinine ratio of ≥30 mg/g | - | - |  | - | - |  |
| **TG and HDL-C counted as:** | Two separate criteria | Two separate criteria | Two separate criteria | Two separate criteria | One combined criterion | Two separate criteria |  |
| Abbreviations: AHA/NHLBI, American Heart Association / National Heart, Lung, and Blood Institute; BMI, Body mass index; BP, Blood pressure; CT, Computed tomography; HDL-C, High-density lipoprotein cholesterol; IDF, International Diabetes Federation; IFG, Impaired fasting glucose; IGT, Impaired glucose tolerance; JCCMS, Japanese Committee of the Criteria for Metabolic Syndrome; JIS, Joint Interim Statement; NCEP ATP III, National Cholesterol Education Program Adult Treatment Panel III; T2DM, Type 2 diabetes mellitus; TG, Triglycerides; WC, Waist circumference; WHO, World Health Organization; WHR, Waist-to-hip ratio. | | | | | | | |

1.	Alberti KG, Zimmet PZ. Definition, diagnosis and classification of diabetes mellitus and its complications. Part 1: diagnosis and classification of diabetes mellitus provisional report of a WHO consultation. *Diabet Med*. Jul 1998;15(7):539-53. doi:<https://dx.doi.org/10.1002/(sici)1096-9136(199807)15:7><539::Aid-dia668>3.0.Co;2-s

2.	APTIII N. Executive Summary of The Third Report of The National Cholesterol Education Program (NCEP) Expert Panel on Detection, Evaluation, And Treatment of High Blood Cholesterol In Adults (Adult Treatment Panel III). *Jama*. May 16 2001;285(19):2486-97. doi:<https://dx.doi.org/10.1001/jama.285.19.2486>

3.	Grundy SM, Cleeman JI, Daniels SR, et al. Diagnosis and management of the metabolic syndrome: an American Heart Association/National Heart, Lung, and Blood Institute Scientific Statement. *Circulation*. Oct 25 2005;112(17):2735-52. doi:<https://dx.doi.org/10.1161/circulationaha.105.169404>

4.	IDF. IDF Consensus Worldwide Definition of the Metabolic Syndrome. 2006. <https://idf.org/about-diabetes/resources/idf-consensus-worldwide-definition-of-the-metabolic-syndrome/>

5.	JCCMS. Definition and the diagnostic standard for metabolic syndrome: Committee to Evaluate Diagnostic Standards for Metabolic Syndrome. *Nihon Naika Gakkai Zasshi*. Apr 10 2005;94(4):794-809.

6.	Alberti KG, Eckel RH, Grundy SM, et al. Harmonizing the metabolic syndrome: a joint interim statement of the International Diabetes Federation Task Force on Epidemiology and Prevention; National Heart, Lung, and Blood Institute; American Heart Association; World Heart Federation; International Atherosclerosis Society; and International Association for the Study of Obesity. *Circulation*. Oct 20 2009;120(16):1640-5. doi:<https://dx.doi.org/10.1161/circulationaha.109.192644>

**Supplementary Table S5 References:**

**Table S6**: Key pertinent study characteristics.

| **Author & year (Ref.)** | **Country** | **Study design** | **Data sources** | **Sample size** | **Sex** | | **Age (Mean ±SD)** | **BMI (Mean ±SD)** | **Ethnicity** | **Diagnostic criteria for MetS** | **Prevalence of MetS (%)** | **OSA Severity (AHI events/hr)** | **Sample size by severity** |
| --- | --- | --- | --- | --- | --- | --- | --- | --- | --- | --- | --- | --- | --- |
| Adar et al. 2019 [61] | Turkey | Cross-sectional | Hospital based | 314 | M | 209 | 51.2 ± 10.9 | 32.7 ± 6.1 | Not specified | NCEP ATP III (2001) | 58.6 | ≥ 5 AHI <15 | 137 |
|  |  |  |  |  | F | 105 |  |  |  |  |  | ≥ 15 AHI < 30 | 97 |
|  |  |  |  |  |  |  |  |  |  |  |  | AHI ≥ 30 | 80 |
| Agrawal et al. 2011 [62] | India | Cross-sectional | Hospital based | 187 | M | 153 | 46.0 ± 8.0 | 33.5 ± 7.0 | Not specified | NCEP ATP III (2001) | 79.0 | ≥ 5 AHI <15 | 41 |
|  |  |  |  |  | F | 34 |  |  |  |  |  | ≥ 15 AHI < 30 | 36 |
|  |  |  |  |  |  |  |  |  |  |  |  | AHI ≥ 30 | 110 |
| Ahbab et al. 2013 [63] | Turkey | Cross-sectional | Hospital based | 44 | M | 25 | 50.32 ± 9.34 | 32.56 ± 6.6 | Not specified | NCEP ATP III (2005) | 63.6 | AHI < 30 | 19 |
|  |  |  |  |  | F | 19 |  |  |  |  |  | AHI ≥ 30 | 25 |
| Akahoshi et al. 2010 [64] | Japan | Cross-sectional | Hospital based | 416 | M | 416 | 52.8 ± 13.7 | 28.0 ± 4.8 | Not specified | National  (JCCMS)(2005) | 52.4 | 5 ≤ AHI < 15 | 32 |
|  |  |  |  |  | F | 0 |  |  |  |  |  | 15 ≤ AHI < 30 | 83 |
|  |  |  |  |  |  |  |  |  |  |  |  | AHI ≥ 30 | 301 |
| Akarsu et al. 2023 [20] | Turkey | Cross-sectional | Hospital based | 73 | M | 49 | 50.3 ± 12.1 | 29.0 ± 5.0 | Not specified | NR | 35.6 | AHI ≥ 5 | 73 |
|  |  |  |  |  | F | 24 |  |  |  |  |  |  |  |
| Akbal et al. 2008 [65] | Turkey | Cross-sectional | Hospital based | 80 | M | 54 | 50.9 ± 9 | 32.6 ± 6.0 | Not specified | NCEP ATP III (2001) | 66.3 | AHI 5-14.9 | 29 |
|  |  |  |  |  | F | 26 |  |  |  |  |  | AHI 15-29.9 | 18 |
|  |  |  |  |  |  |  |  |  |  |  |  | AHI ≥ 30 | 33 |
| Aksu et al. 2015 [66] | Turkey | Cross-sectional | Hospital based | 41 | M | 34 | 46.9 ± 11.5 | 32.5 ± 6.4 | Not specified | NCEP ATP III (2005) | 58.5 | AHI 5-15 | 12 |
|  |  |  |  |  | F | 7 |  |  |  |  |  | AHI 15-30 | 12 |
|  |  |  |  |  |  |  |  |  |  |  |  | AHI ≥ 30 | 17 |
| Angelico et al. 2010 [67] | Italy | Cross-sectional | Hospital based | 178 | M | 142 | 53.7 ± 11.6 | 32.1 ± 6.0 | Not specified | NCEP ATP III (2001) | 53.9 | AHI 5-14 | 54 |
|  |  |  |  |  | F | 36 |  |  |  |  |  | AHI 15-29 | 51 |
|  |  |  |  |  |  |  |  |  |  |  |  | AHI ≥ 30 | 73 |
| Apaydin et al. 2013 [68] | Turkey | Cross-sectional | Hospital based | 67 | M | 53 | 51.8 ± 10.2 | 31.6 ± 5.9 | Not specified | Harmonizing criteria (2009) | 49.0 | 5 ≥ AHI < 30 | 27 |
|  |  |  |  |  | F | 14 |  |  |  |  |  | AHI ≥ 30 | 40 |
| Archontogeorgis et al. 2018 [69] | Greece | Cross-sectional | Hospital based | 107 | M | 88 | 53.4 ± 12.9 | 36.5 ± 7.9 | Caucasian | Harmonizing criteria (2009) | 51.4 | AHI ≥ 5 | 107 |
|  |  |  |  |  | F | 19 |  |  |  |  |  |  |  |
| Archontogeorgis et al. 2020 [70] | Greece | Cross-sectional | Hospital based | 163 | M | 138 | 60.8 ± 9.9 | 36.5 ± 6.4 | Not specified | Harmonizing criteria (2009) | 38.0 | AHI ≥ 5 | 163 |
|  |  |  |  |  | F | 25 |  |  |  |  |  |  |  |
| Baburao et al. 2022 [71] | India | Quasi-experimental | Hospital based | 145 | M | 87 | 51.6 ± 8.6 | 32.9 ± 5.9 | Not specified | IDF (2005) | 73.1 | AHI > 5 | 145 |
|  |  |  |  |  | F | 58 |  |  |  |  |  |  |  |
| Banawan et al. 2017 [72] | Egypt | Cross-sectional | Hospital based | 28 | M | 12 | 52.5 ± 9.5 | 36.2 ± 9.4 | Not specified | NCEP ATP III (2001) | 50.0 | AHI 5-15 | 6 |
|  |  |  |  |  | F | 16 |  |  |  |  |  | AHI 15-30 | 8 |
|  |  |  |  |  |  |  |  |  |  |  |  | AHI > 30 | 14 |
| Barceló et al. 2011 [74] | Spain | Case-Control | Hospital based | 119 | M | 88 | 46.0 ± 12.0 | 28.0 ± 4.0 | Not specified | NCEP ATP III (2005) | 38.0 | AHI ≥ 10 | 119 |
|  |  |  |  |  | F | 31 |  |  |  |  |  |  |  |
| Barceló et al. 2016 [73] | Spain | Case-Control | Hospital based | 38 | M | 21 | 50.0 ± 12.0 | 28.0 ± 6.0 | Not specified | Harmonizing criteria (2009) | 37.0 | AHI ≥ 10 | 38 |
|  |  |  |  |  | F | 17 |  |  |  |  |  |  |  |
| Barreiro et al. 2013 [75] | Spain | Cross-sectional | Hospital based | 116 | M | 90 | NR | 32.8 ± 5.4 | Not specified | NCEP ATP III (2001) | 68.2 | 15 ≤ AHI < 30 | 41 |
|  |  |  |  |  | F | 26 |  |  |  |  |  | AHI ≥ 30 | 75 |
| Basoglu et al. 2011 [76] | Turkey | Cross-sectional | Hospital based | 36 | M | 23 | 50.0 ± 19.7 | 33.5 ± 5.7 | Not specified | NCEP ATP III (2001) | 47.2 | AHI > 5 | 36 |
|  |  |  |  |  | F | 13 |  |  |  |  |  |  |  |
| Bhushan et al. 2010 [77] | India | Case-Control | Hospital based | 121 |  | 101 | 45.1 ± 9.4 | 31.5 ± 4.3 | Not specified | NCEP ATP III (2001) | 67.8 | AHI ≥ 10 | 121 |
|  |  |  |  |  | F | 20 |  |  |  |  |  |  |  |
| Bingol et al. 2019 [78] | Turkey | Cross-sectional | Hospital based | 146 | M | 65 | 49.8 ± 10.7 | 40.3 ± 4.9 | Not specified | WHO (1998) | 61.6 | AHI 5-29 | 76 |
|  |  |  |  |  | F | 81 |  |  |  |  |  | AHI ≥ 30 | 70 |
| Bonsignore et al. 2012 [79] | Spain | Cross-sectional | Hospital based | 529 | M | 424 | 51.3 ± 12.8 | 30.8 ± 6.0 | Caucasian | Harmonizing criteria (2009) | 51.2 | AHI ≥ 10 | 529 |
|  |  |  |  |  |  | 105 |  |  |  |  |  |  |  |
| Bozkurt et al. 2016 [80] | Turkey | Case-control | Hospital based | 158 | M | 80 | 46.6 ± 8.2 | 29.2 ± 4.6 | Not specified | WHO (1998) | 30.0 | AHI ≥ 5 | 158 |
|  |  |  |  |  | F | 78 |  |  |  | NCEP ATP III (2001) | 35.6 |  |  |
|  |  |  |  |  |  |  |  |  |  | IDF (2005) | 44.4 |  |  |
|  |  |  |  |  |  |  |  |  |  | At least one definition | 72.8 |  |  |
| Breuer et al. 2011 [81] | Germany | Cross-sectional | Hospital based | 360 | NR | NR | 56.0 ± 208.7 | 32.0 ± 151.8 | Not specified | NCEP ATP III (2005) | 47.0 | AHI ≥ 5 | 360 |
|  |  |  |  |  |  |  |  |  |  | German definition of the “Metabolic Vascular Syndrome – MVS” | 58.0 |  |  |
|  |  |  |  |  |  |  |  |  |  | IDF (2005) | 51.0 |  |  |
| Chaudhary et al. 2021 [82] | India | Cross-sectional | Hospital based | 502 | M | 357 | 51.88 ± 12.2 | 31.6 ± 11.1 | Not specified | NCEP ATP III (2005) | 72.7 | AHI 5-14.9 | 36 |
|  |  |  |  |  | F | 145 |  |  |  |  |  | AHI 15-29.9 | 77 |
|  |  |  |  |  |  |  |  |  |  |  |  | AHI > 30 | 389 |
| Chen et al. 2016 [83] | China | Cross-sectional | Hospital based | 361 | M | 282 | 49.6 ± 13.1 | 26.8 ± 5.2 | Not specified | NCEP ATP III (2005) | 61.5 | AHI 5-14.9 | 67 |
|  |  |  |  |  | F | 79 |  |  |  |  |  | AHI 15-29.9 | 89 |
|  |  |  |  |  |  |  |  |  |  |  |  | AHI > 30 | 205 |
| Coughlin et al. 2004 [84] | United Kingdom | Case-Control | Hospital based | 61 | M | 61 | 49.6 ± 9.4 | 35.8 ± 7.0 | Not specified | NCEP ATP III (2001) | 87.0 | AHI > 15 | 61 |
|  |  |  |  |  | F | 0 |  |  |  |  |  |  |  |
| Coughlin et al. 2007 [85] | United Kingdom | Randomized Controlled trial | Hospital based | 68 | M | 68 | 49.0 ± 8.3 | 36.1 ± 7.6 | Caucasian | NCEP ATP III (2001) | 82.4 | AHI > 15 | 68 |
|  |  |  |  |  | F | 0 |  |  |  |  |  |  |  |
| Desplan et al. 2014 [86] | France | Randomized Controlled trial | Hospital based | 22 | NR | NR | NR | 30.6 ± 3.0 | Not specified | IDF (2005) | 90.9 | AHI > 15 | 22 |
| Dobrowolski 2021 [87] | Poland | Cross-sectional | Hospital based | 94 | M | 69 | 57.3 ± 10.2 | 33.5 ± 4.7 | Not specified | NCEP ATP III (2001) | 88.3 | AHI ≥ 15  or AHI ≥ 5 | 94 |
|  |  |  |  |  | F | 25 |  |  |  |  |  |  |  |
| Domagala-Kulawik et al. 2015 [88] | Poland | Cross-sectional | Hospital based | 48 | M | 33 | 58.5 ± 11.5 | 31.68 ± 6.8 | Not specified | IDF (2005) | 56.0 | AHI ≥ 5 | 48 |
|  |  |  |  |  | F | 15 |  |  |  |  |  |  |  |
| Drager et al. 2010 [89] | Brazil | Cross-sectional | Hospital based | 55 | M | 30 | 51.0 ± 10.0 | 31.0 ± 4.7 | Majority Whites (57%) | NCEP ATP III (2005) | 86.0 | AHI > 5 | 55 |
|  |  |  |  |  | F | 25 |  |  |  |  |  |  |  |
| Du et al. 2024 [90] | China | Cross-sectional | Hospital based | 112 | M | 100 | 43.5 ± 11.6 | 27.10 ± 4.7 | Not specified | IDF (2005) | 45.5 | AHI ≥ 5 | 112 |
|  |  |  |  |  | F | 12 |  |  |  |  |  |  |  |
| Dursunoglu et al. 2006 [91] | Turkey | Cross-sectional | Hospital based | 87 | M | 54 | 48.9 ± 7.0 | 30.1 ± 3.5 | Not specified | NCEP ATP III (2001) | 54.0 | AHI < 30 | 46 |
|  |  |  |  |  | F | 33 |  |  |  |  |  | AHI ≥ 30 | 41 |
| Florczak et al. 2013 [92] | Poland | Cross-sectional | Hospital based | 147 | M | 99 | 49.9 ± 9.8 | 31.0 ± 4.4 | Not specified | NR | 71.4 | AHI > 5 | 147 |
|  |  |  |  |  | F | 48 |  |  |  |  |  |  |  |
| Fu et al. 2017 [93] | China | Cross-sectional | Hospital based | 1926 | M | 1926 | NR | NR | Han Chinese | NCEP ATP III (2005) | 55.2 | AHI 5-15 | 367 |
|  |  |  |  |  | F | 0 |  |  |  |  |  | AHI 15-30 | 352 |
|  |  |  |  |  |  |  |  |  |  |  |  | AHI > 30 | 1207 |
| Gasa et al. 2011 [94] | Spain | Cross-sectional | Hospital based | 115 | M | 33 | 44.9 ± 9.6 | 46.7 ± 6.1 | Not specified | Harmonizing criteria (2009) | 70.0 | AHI 15-30 | 29 |
|  |  |  |  |  | F | 82 |  |  |  |  |  | AHI 30-50 | 42 |
|  |  |  |  |  |  |  |  |  |  |  |  | AHI > 50 | 43 |
| Georgoulis et al. 2020 [95] | Greece | Randomized Controlled trial | Hospital based | 187 | M | 141 | 49.0 ± 10.0 | 35.6 ± 6.0 | Not specified | Harmonizing criteria (2009) | 62.0 | AHI ≥ 15 | 187 |
|  |  |  |  |  | F | 46 |  |  |  |  |  |  |  |
| Gothi et al. 2023 [34] | India | Cross-sectional | Hospital based | 249 | M | 177 | 51.4 ± 10.9 | 32.6 ± 6.2 | Not specified | NCEP ATP III (2005) | 40.16 | AHI ≥ 5 | 249 |
|  |  |  |  |  | F | 72 |  |  |  |  |  |  |  |
| Gu et al. 2021 [96] | China | Cross-sectional | Hospital based | 2557 | M | 2141 | 43.1±14.1 | 26.9 ± 3.5 | Han Chinese | NCEP ATP III (2005) | 49.1 | AHI ≥ 5 | 2557 |
|  |  |  |  |  | F | 416 |  |  |  |  |  |  |  |
| Hermans et al. 2009 [98] | Belgium | Cross-sectional | Hospital based | 43 | M | 43 | 63.0 ± 10.0 | 34.2 ± 6.5 | North-Caucasians | NCEP ATP III (2005) | 97.0 | AHI ≥ 5 | 43 |
|  |  |  |  |  | F | 0 |  |  |  |  |  |  |  |
| Hermans et al. 2013 [97] | Belgium | Cross-sectional | Hospital based | 25 | M | 0 | 65.0 ± 9.0 | 38.7 ± 6.5 | White Caucasians (83%) | Harmonizing criteria (2009) | 100.0 | AHI ≥ 5 | 25 |
|  |  |  |  |  | F | 25 |  |  |  |  |  |  |  |
| Hermans et al. 2014 [99] | Belgium | Cross-sectional | Hospital based | 129 | M | 100 | 63.5 ± 9.8 | 35.0 ± 6.3 | Belgians/Maghrebians (not representative of all participants analysed) | Harmonizing criteria (2009) | 96.1 | AHI ≥ 5 | 129 |
|  |  |  |  |  | F | 29 |  |  |  |  |  |  |  |
| Hirotsu et al. 2018 [21] | Brazil | Cohort | Population based | 1046 | NR | NR | NR | NR | White/Caucasians (88%) | Harmonizing criteria (2009) | 17.1 | AHI ≥ 5 ≤ 15 | 609 |
|  | Switzerland |  |  |  |  |  |  |  |  |  |  | AHI ≥ 15 | 437 |
| Huang et al. 2015 [100] | China | Cross-sectional | Hospital based | 175 | M | 126 | 43.8 ± 11.0 | 27.5 ± 4.5 | Not specified | NCEP ATP III (2005) | 62.3 | AHI ≥ 30 | 175 |
|  |  |  |  |  | F | 49 |  |  |  |  |  |  |  |
| Incalzi et al. 2014 [101] | Italy | Quasi-experimental | Hospital based | 50 | M | 37 | 63.1 ± 13.1 | 33.4 ± 5.6 | Not specified | NR | 44.0 | AHI ≥ 10 | 50 |
|  |  |  |  |  | F | 13 |  |  |  |  |  |  |  |
| Iren et al. 2011 [102] | Romania | Cross-sectional | Hospital based | 34 | M | 22 | 45.9 ± 11.5 | 33.8 ± 5.3 | Not specified | NR | 79.4 | AHI > 5 | 34 |
|  |  |  |  |  | F | 12 |  |  |  |  |  |  |  |
| Izumi et al. 2016 [103] | Brazil | Cross-sectional | Hospital based | 36 | M | 36 | 47.7 ± 7.1 | 33.4 ± 4.0 | Not specified | Harmonizing criteria (2009) | 50.0 | AHI 5-14.9 | 8 |
|  |  |  |  |  | F | 0 |  |  |  |  |  | AHI ≥ 15 | 28 |
| Joshi et al. 2016 [104] | India | Cross-sectional | Hospital based | 81 | M | 47 | 51.4 ± 9.4 | 27.9 ± 3.3 | Not specified | NCEP ATP III (2005) | 75.3 | 5 = AHI <15 | 19 |
|  |  |  |  |  | F | 34 |  |  |  |  |  | 15> AHI <30 | 24 |
|  |  |  |  |  |  |  |  |  |  |  |  | AHI = 30 | 18 |
| Kaddah et al. 2024 [105] | Egypt | Cross-sectional | Hospital based | 80 | M | 33 | 53.0 ± 12.0 | 41.9 ± 9.6 | Not specified | IDF (2005) | 66.3 | AHI ≥ 5 | 80 |
|  |  |  |  |  | F | 47 |  |  |  |  |  |  |  |
| Kanbay et al. 2009 [106] | Turkey | Cross-sectional | Hospital based | 277 | M | 206 | 49.8 ± 10.3 | 31.1 ± 5.7 | Not specified | NCEP ATP III (2001) | 39.7 | AHI 5-15 | 85 |
|  |  |  |  |  | F | 71 |  |  |  |  |  | AHI ≥ 30 | 192 |
| Kawaguchi et al. 2010 [107] | Japan | Cross-sectional | Hospital based | 189 | M | 151 | 53.8 ± 14.3 | 27.3 ± 4.8 | Not specified | NCEP ATP III (2005) | 22.8 | AHI ≥ 5 | 189 |
|  |  |  |  |  | F | 38 |  |  |  |  |  |  |  |
| Kim et al. 2017 [108] | South Korea | Cohort | Population based | 462 | M | 294 | 58.1 ± 7.6 | 25.5 ± 3.0 | Not specified | NCEP ATP III (2001) | 34.2 | AHI ≥ 10 | 462 |
|  |  |  |  |  | F | 168 |  |  |  |  |  |  |  |
| Koo et al. 2020 [109] | South Korea | Cross-sectional | Hospital based | 52 | NR | NR | NR | NR | Not specified | NCEP ATP III (2005) | 51.9 | 5 ≤ AHI < 15 | 27 |
|  |  |  |  |  |  |  |  |  |  |  |  | 15 ≤ AHI < 30 | 15 |
|  |  |  |  |  |  |  |  |  |  |  |  | AHI ≥ 30 | 10 |
| Kraiwattanapong et al. 2025 [110] | Thailand | Cross-sectional | Hospital based | 377 | M | 242 | 48.2, 46 (Mean, Median) | NR | Not specified | NR | 14.9 | AHI ≥ 5 | 377 |
|  |  |  |  |  | F | 135 |  |  |  |  |  |  |  |
| Kumor et al. 2013 [31] | Poland | Cross-sectional | Hospital based | 173 | M | 155 | 53.9 ± 9.3 | 31.8 ± 5.0 | Not specified | NCEP ATP III (2001) | 56.0 | AHI ≥ 5 | 173 |
|  |  |  |  |  | F | 18 |  |  |  | IDF (2005) | 69.0 |  |  |
| Lam et al. 2006 [30] | China | Cross-sectional | Community based | 95 | M | 64 | 45.1 ± 6.9 | 27.2 ± 3.3 | Not specified | NCEP ATP III (2001) | 58.0 | AHI 5-14.9 | 48 |
|  |  |  |  |  |  |  |  |  |  |  |  | AHI 15-29.9 | 27 |
|  |  |  |  |  | F | 31 |  |  |  |  |  | AHI ≥ 30 | 20 |
| Lee et al. 2016 [111] | Taiwan | Cross-sectional | Hospital based | 30 | M | 27 | 39.2 ± 7.8 | 25.5 ± 1.4 | Not specified | NCEP ATP III (2005) | 30.0 | AHI ≥ 5 | 30 |
|  |  |  |  |  | F | 3 |  |  |  |  |  |  |  |
| Lee et al. 2017 [112] | South Korea | Cross-sectional | Hospital based | 1732 | M | 1482 | 53.9 ± 9.1 | 25.2 ± 1.4 | Not specified | NCEP ATP III (2005) | 29.2 | AHI 5 to < 15 | 584 |
|  |  |  |  |  | F | 250 |  |  |  |  |  | AHI 15 to < 30 | 516 |
|  |  |  |  |  |  |  |  |  |  |  |  | AHI ≥ 30 | 632 |
| Liu et al. 2021 [36] | China | Cohort | Hospital based | 1157 | M | 704 | 66.4 ± 6.7 | 26.5 ± 1.3 | Not specified | NCEP ATP III (2005) | 60.8 | AHI 5-14.9 | 289 |
|  |  |  |  |  | F | 453 |  |  |  |  |  | AHI 15-30 | 347 |
|  |  |  |  |  |  |  |  |  |  |  |  | AHI > 30 | 521 |
| Li et al. 2020 [113] | China | Cross-sectional | Hospital based | 3411 | M | 3007 | 42.5 ± 12.8 | 27.5 ± 3.2 | Han Chinese | NCEP ATP III (2005) modified for Chinese ethnicity specific | 60.9 | AHI 5-30 | 831 |
|  |  |  |  |  | F | 404 |  |  |  |  |  | AHI > 30 | 2580 |
| Lubrano et al. 2012 [114] | Italy | Cross-sectional | Hospital based | 104 | Male | 42 | 50.2 ± 12.3 | 43.4 ± 9.5 | Caucasian | Harmonizing criteria (2009) | 65.4 | AHI 5-14.9 | 42 |
|  |  |  |  |  | F | 62 |  |  |  |  |  | AHI 15-29.9 | 26 |
|  |  |  |  |  |  |  |  |  |  |  |  | AHI ≥ 30 | 36 |
| Makino et al. 2009 [115] | Japan | Cross-sectional | Hospital based | 157 | M | 157 | 52.6 ± 12.8 | 26.4 ± 4.2 | Not specified | National (JCCMS)(2005) | 31.2 | ≥ 5 AHI < 20 | 40 |
|  |  |  |  |  | F | 0 |  |  |  |  |  | ≥ 20AHI < 40 | 64 |
|  |  |  |  |  |  |  |  |  |  |  |  | AHI ≥ 40 | 53 |
| Mano et al. 2023 [116] | Japan | Cross-sectional | Hospital based | 836 | M | 601 | 57.6 ± 14.0 | 25.1 ± 10.3 | Not specified | National (JCCMS) (2005) | 30.0 | 5 ≤ AHI ≤ 30 | 836 |
|  |  |  |  |  | F | 235 |  |  |  |  |  |  |  |
| Mariani et al. 2013 [117] | Italy | Cross-sectional | Hospital based | 115 | M | 56 | 53.6 ± 9.5 | 36.3 ± 4.6 | Not specified | NR | 55.65 | AHI 5-14 | 50 |
|  |  |  |  |  | F | 59 |  |  |  |  |  | AHI 15-29 | 33 |
|  |  |  |  |  |  |  |  |  |  |  |  | AHI ≥ 30 | 32 |
| Mazzuca et al. 2014 [118] | Spain | Cross-sectional | Hospital based | 473 | M | 388 | 51.9 ± 12.4 | 31.6 ± 6.2 | Caucasian | Harmonizing criteria (2009) | 46.9 | AHI > 10 ≤ 30 | 144 |
|  |  |  |  |  | F | 85 |  |  |  |  |  | AHI ≥ 30 | 329 |
| McArdle et al. 2006 [119] | Australia | Case-control | Hospital based | 21 | M | 21 | 46 ± 10.2 | 28.4 ± 3.4 | Not specified | NR | 23.8 | AHI > 15 | 21 |
|  |  |  |  |  | F | 0 |  |  |  |  |  |  |  |
| Murase et al. 2013 [120] | Japan | Cross-sectional | Hospital based | 87 | M | 60 | 56.8 ± 13.1 | 26.2 ± 5.9 | Not specified | National (JCCMS) (2005) | 36.8 | 5 ≤ AHI < 15 | 37 |
|  |  |  |  |  | F | 27 |  |  |  |  |  | 15 ≤ AHI < 30 | 24 |
|  |  |  |  |  |  |  |  |  |  |  |  | AHI ≥ 30 | 26 |
| Nakanishi-Minami et al. 2012 [121] | Japan | Cross-sectional | Hospital based | 39 | M | 39 | 52.0 ± 6.2 | 27.8 ± 3.7 | Not specified | National (JCCMS) (2005) | 41.0 | AHI ≥ 5 to < 15 | 7 |
|  |  |  |  |  | F | 0 |  |  |  |  |  | AHI ≥ 15 to < 30 | 11 |
|  |  |  |  |  |  |  |  |  |  |  |  | AHI ≥ 30 | 21 |
| Nerfeldt et al. 2008 [122] | Sweden | Quasi-experimental | Hospital based | 33 | M | 24 | 52.0 ± 9.3 | 40.0 ± 4.3 | Not specified | IDF (2005) | 81.8 | AHI ≥ 10 | 33 |
|  |  |  |  |  | F | 9 |  |  |  |  |  |  |  |
| Ozol et al. 2011 [123] | Turkey | Cross-sectional | Hospital based | 105 | M | 75 | 52.3 ± 12.5 | 30.8 ± 6.4 | Not specified | NCEP ATP III (2001) | 23.8 | AHI 5-14.9 | 27 |
|  |  |  |  |  | F | 30 |  |  |  |  |  | AHI 15-29.9 | 24 |
|  |  |  |  |  |  |  |  |  |  |  |  | AHI ≥ 30 | 54 |
| Papanas et al. 2010 [124] | Greece | Cross-sectional | Hospital based | 53 | M | 53 | 46.1 ± 10.9 | 34.1 ± 7.4 | Caucasian | NCEP ATP III (2001) | 71.7 | AHI > 5 | 53 |
|  |  |  |  |  | F | 0 |  |  |  |  |  |  |  |
| Parish et al. 2007 [125] | United States | Cross-sectional | Hospital based | 174 | M | 115 | 63.4 ± 12.8 | 32.2 ± 7.6 | Not specified | NCEP ATP III (2001) | 56.0 | AHI 5-14 | 57 |
|  |  |  |  |  | F | 59 |  |  |  |  |  | AHI 15-29 | 57 |
|  |  |  |  |  |  |  |  |  |  |  |  | AHI ≥ 30 | 60 |
| Pedrosa et al. 2011 [126] | Brazil | Cross-sectional | Hospital based | 80 | M | 42 | 55.0 ± 8.0 | 32.9 ± 6.7 | White (48%) | NCEP ATP III (2005) | 75.0 | AHI ≥ 15 | 80 |
|  |  |  |  |  | F | 38 |  |  |  |  |  |  |  |
| Peled et al. 2007 [127] | Israel | Cross-sectional | Hospital based | 89 | M | 89 | 55.1 ± 13.2 | 30.0 ± 5.3 | Not specified | NCEP ATP III (2001) | 25.8 | 5 ≤ AHI < 15 | 9 |
|  |  |  |  |  | F | 0 |  |  |  |  |  | 15 ≤ AHI < 30 | 27 |
|  |  |  |  |  |  |  |  |  |  |  |  | AHI ≥ 30 | 53 |
| Peng et al. 2024 [128] | China | Cross-sectional | Population based | 3779 | M | 3272 | 44.4 ± 2 | 27.6± 5.9 | Han Chinese | NCEP ATP III (2005) | 59.5 | AHI 5.0-14.9 | 256 |
|  |  |  |  |  | F | 507 |  |  |  |  |  | AHI 15.0-29.9 | 938 |
|  |  |  |  |  |  |  |  |  |  |  |  | AHI ≥ 30.0 | 2585 |
| Pilkauskaite et al. 2014 [129] | Lithuania | Cross-sectional | Hospital based | 65 | M | 65 | 40.4 ± 8.3 | 31.6 ± 5.7 | Not specified | NCEP ATP III (2005) | 52.3 | AHI ≥ 5 | 65 |
|  |  |  |  |  | F | 0 |  |  |  |  |  |  |  |
| Prejbisz et al. 2013 [130] | Poland | Cross-sectional | Hospital based | 92 | M | 74 | 50.9 ± 9.1 | 31.9 ± 4.3 | Not specified | NCEP ATP III (2001) | 77.2 | AHI > 15 | 92 |
|  |  |  |  |  | F | 18 |  |  |  |  |  |  |  |
| Qamar et al. 2017 [131] | Pakistan | Case-Control | Hospital based | 50 | M | 30 | 49.4 ± 8.0 | 31.8 ± 6.7 | Not specified | NCEP-ATP III criteria in which the ethnic specific BMI | 76.0 | AHI 5-15 | 6 |
|  |  |  |  |  | F | 20 |  |  |  |  |  | AHI 15-30 | 14 |
|  |  |  |  |  |  |  |  |  |  |  |  | AHI ≥ 30 | 18 |
| Sachdev et al. 2009 [132] | India | Case-control | Hospital based | 48 | M | 39 | 50.3 ± 7.4 | 30.1 ± 1.2 | Not specified | IDF (2005) Asian Definition | 77.0 | RDI ≥ 5 | 48 |
|  |  |  |  |  | F | 9 |  |  |  |  |  |  |  |
| Salord et al. 2016 [133] | Spain | Randomized Controlled Trial | Hospital based | 80 | M | 22 | 46.7 ± 9.1 | 47.4 ± 6.1 | Not specified | Harmonizing criteria (2009) | 78.8 | AHI > 30 | 80 |
|  |  |  |  |  | F | 58 |  |  |  |  |  |  |  |
| Sasanabe et al. 2006 [134] | Japan | Cross-sectional | Hospital based | 819 | M | 719 | 49.2 ± 13.5 | 27.7 ± 5.6 | Not specified | National (JCCMS) (2005) | 47.4 | AHI: 5–14.9 | 179 |
|  |  |  |  |  | F | 100 |  |  |  |  |  | AHI: 15–29.9 | 178 |
|  |  |  |  |  |  |  |  |  |  |  |  | AHI ≥ 30 | 462 |
| Seetho et al. 2014A [135] | United Kingdom | Cross-sectional | Hospital based | 47 | M | 24 | 47.0 ± 9.0 | 42.0 ± 7.0 | White European | NCEP ATP III (2001) | 60.0 | AHI ≥ 5 | 47 |
|  |  |  |  |  | F | 23 |  |  |  |  |  |  |  |
| Seetho et al. 2014C [137] | United Kingdom | Case-Control | Hospital based | 61 | M | 30 | 47.9 ± 9.0 | 43.0 ± 8.0 | White European | NCEP ATP III (2001) | 57.0 | AHI ≥ 5 | 61 |
|  |  |  |  |  | F | 31 |  |  |  |  |  |  |  |
| Seetho et al. 2015B [136] | United Kingdom | Case-Control | Hospital based | 27 | M | 15 | 49.0 ± 7.0 | 43.0 ± 7.0 | White European | NCEP ATP III (2001) | 52.0 | AHI ≥ 5 | 27 |
|  |  |  |  |  | F | 12 |  |  |  |  |  |  |  |
| Sharma et al. 2010 [138] | India | Cross-sectional | Community based | 94 | NR | NR | NR | NR | Not specified | NCEP ATP III (2001) | 74.4 | AHI ≥ 5 | 94 |
|  |  |  |  |  |  |  |  |  |  |  |  |  |  |
| Shina et al. 2006 [139] | Japan | Cross-sectional | Hospital based | 94 | M | 88 | 52.0 ± 1.0 | 28.1 ± 0.5 | Not specified | NCEP ATP III (2005) | 43.6 | AHI ≥ 15 | 94 |
|  |  |  |  |  | F | 6 |  |  |  |  |  |  |  |
| Silva et al. 2018 [140] | Brazil | Cross-sectional | Hospital based | 85 | M | 45 | 47.0 ± 9.0 | 28.0 ± 4.0 | Not specified | NCEP ATP III (2005) | 32.0 | ≥ 5 AHI ≤ 15 | 85 |
|  |  |  |  |  | F | 40 |  |  |  |  |  |  |  |
| Singh et al. 2020 [141] | India | Cross-sectional | Hospital based | 50 | M | 41 | 42.5 ± 11.2 | 27.0 ± 2.3 | Not specified | NCEP ATP III (2001) | 62.0 | AHI ≥ 5 | 50.0 |
|  |  |  |  |  | F | 9 |  |  |  |  |  |  |  |
| Solelhac et al. 2025 [142] | India | Cross-sectional | Population based | 293 | M | 186 | 45.0 ± 12.6 | 26.6 ± 4.5 | Not specified | NCEP ATP III | 55.2 | AHI ≥ 15 | 293 |
|  |  |  |  |  | F | 107 |  |  |  |  |  |  |  |
|  | Switzerland |  |  | 710 | M | 474 | 62.7 ± 12.3 | 26.9 ± 3.6 |  |  | 46.8 |  | 710 |
|  |  |  |  |  | F | 236 |  |  |  |  |  |  |  |
| Srivastava et al. 2022 [143] | India | Cross-sectional | Hospital based | 94 | NR | NR | NR | NR | Not specified | NCEP ATP III (2001) | 63.8 | AHI 5-15 | 18 |
|  |  |  |  |  |  |  |  |  |  |  |  | AHI 16-30 | 26 |
|  |  |  |  |  |  |  |  |  |  |  |  | AHI > 30 | 50 |
| Tancˇ ic´-Gajic et al. 2021 [144] | Serbia | Cross-sectional | Hospital based | 104 | M | 104 | 41.8 ± 11.9 | 45.7 ± 7.4 | Not specified | Harmonizing criteria (2009) | 77.9 | AHI < 15 | 20 |
|  |  |  |  |  |  |  |  |  |  |  |  | AHI 15-29.9 | 17 |
|  |  |  |  |  | F | 0 |  |  |  |  |  | AHI ≥ 30 | 67 |
| Theorell-Haglöw et al. 2010 [156] | Sweden | Cross-sectional | Population based | 135 | M | 0 | 55.8 ± 8.8 | 28.7 ± 5.5 | Not specified | NCEP ATP III (2005) | 44.4 | AHI ≥ 15 | 135 |
|  |  |  |  |  | F | 135 |  |  |  |  |  |  |  |
| Tkacova et al. 2008 [145] | Slovakia | Cross-sectional | Hospital based | 70 | M | 49 | 49.8 ± 12.2 | 32.4 ± 7.1 | Not specified | IDF (2005) | 62.9 | AHI ≥ 5 to < 30 | 39 |
|  |  |  |  |  | F | 21 |  |  |  |  |  | AHI ≥ 30 | 31 |
| Usui et al. 2011 [146] | Japan | Cross-sectional | Hospital based | 660 | M | 558 | 51.1 ± 14.2 | 26.6 ± 4.7 | Not specified | NCEP ATP III (2005) | 43.9 | 5 ≤ AHI < 30 | 301 |
|  |  |  |  |  | F | 102 |  |  |  |  |  | AHI ≥ 30 | 359 |
| Utriainen et al. 2013 [147] | Finland | Cross-sectional | Hospital based | 70 | M | 45 | 67.7 ± 9.0 | 27.0 ± 4.2 | Not specified | NCEP ATP III (2005) | 64.3 | AHI 5-15 | 23 |
|  |  |  |  |  | F | 25 |  |  |  |  |  | AHI 15-30 | 23 |
|  |  |  |  |  |  |  |  |  |  |  |  | AHI > 30 | 24 |
| Uyar et al. 2013 [148] | Turkey | Cross-sectional | Hospital based | 674 | M | 475 | 50.8 ± 11.7 | 34.4 ± 7.4 | Not specified | NCEP ATP III (2005) | 58.0 | AHI ≥ 5 | 674 |
|  |  |  |  |  | F | 199 |  |  |  |  |  |  |  |
| Vekic et al. 2016 [149] | Slovakia | Cross-sectional | Hospital based | 140 | M | 98 | 51.3 ± 11.1 | 33.0 ± 5.7 | Not specified | Harmonizing criteria (2009) | 71.4 | AHI 5 and < 30 | 75 |
|  |  |  |  |  | F | 42 |  |  |  |  |  |  |  |
|  |  |  |  |  |  |  |  |  |  |  |  | AHI ≥ 30 | 65 |
| Wang et al. 2024 [150] | China | Cross-sectional | Hospital based | 231 | M | 191 | 44.3 ± 15.3 | 27.5 ± 3.7 | Not specified | NR | 55.8 | 5 ≤ AHI ≤ 30 | 105 |
|  |  |  |  |  | F | 40 |  |  |  |  |  | AHI > 30 | 126 |
| Wu et al. 2015 [151] | Taiwan | Cross-sectional | Hospital based | 162 | M | 162 | 45.6 ± 7.3 | 28.6 ± 4.2 | Not specified | Criteria proposed by the Taiwan Health Promotion Administration, Ministry of Health and Welfare (2007) | 65.4 | AHI 5–14 | 61 |
|  |  |  |  |  | F | 0 |  |  |  |  |  | AHI 15-30 | 56 |
|  |  |  |  |  |  |  |  |  |  |  |  | AHI > 30 | 45 |
| Zhang et al. 2014 [152] | China | Cross-sectional | Hospital based | 108 | M | 78 | 52.1 ± 12.2 | 27.1 ± 3.4 | Not specified | NR | 19.4 | AHI | 56 |
|  |  |  |  |  | F | 30 |  |  |  |  |  | AHI ≥ 15 | 52 |
| Zhang et al. 2022 [153] | China | Cross-sectional | Hospital based | 291 | M | 240 | 44.1 ± 11.6 | 28.4 ± 4.23 | Not specified | Chinese Diabetic Society Guideline (2020) | 45.4 | AHI ≥ 15 | 291 |
|  |  |  |  |  | F | 51 |  |  |  |  |  |  |  |
| Zhao et al. 2019 [154] | China | Cross-sectional | Community based | 1408 | M | 1178 | 43.4 ± 13.6 | 26.9 ± 3.4 | Han Chinese | NCEP ATP III (2001) | 27.9 | AHI 5-14 | 282 |
|  |  |  |  |  | F | 230 |  |  |  |  |  | AHI 15-29 | 320 |
|  |  |  |  |  |  |  |  |  |  |  |  | AHI ≥ 30 | 806 |
| Zhou et al. 2020 [155] | China | Cross-sectional | Hospital based | 28 | NR | NR | NR | NR | Not specified | IDF (2005) | 53.5 | AHI ≥ 5 | 28 |

Abbreviations: AHI, apnoea-hypopnea index; BMI, body mass index; F, Female; IDF, International Diabetes Federation; JCCMS, Japanese Committee of the Criteria for Metabolic Syndrome; M, Male; MetS, metabolic syndrome; NCEP ATP III: National Cholesterol Education Program Adult Treatment Panel III, Third Report of the Expert Panel on Detection, Evaluation, and Treatment of High Blood Cholesterol in Adults; NR, not reported; OSA, obstructive sleep apnoea; SD, standard deviation; WHO, World Health Organization.

**Supplementary Figure S1.** Funnel plot showing publication bias.

**Supplementary Figure S2.** Traffic light plot presenting the assessments for the six domains of the applied Risk of Bias Assessment Tool for Non-randomised Studies (RoBANS) for each of the included studies.

**Supplementary Figure S3.** Tree-plot depicting heterogeneity decomposition across three meta-analysis levels of the applied model.

**Supplementary Figure S4.** Sensitivity analysis plot.

**Supplementary Figure S5.** Baujat plot showing the studies influencing the heterogeneity

**Supplementary Figure S6.** Meta-regression of BMI and prevalence of MetS in patients with OSA. Each bubble represents an individual effect size derived from eligible studies with complete data for age, BMI, and male sex proportion. Bubble size reflects the study weight- larger bubble indicates more precise estimates (i.e., larger sample sizes or narrower confidence intervals), which contribute more to the meta-regression. The solid line is the fitted meta-regression line with the shaded region denoting the 95% confidence interval. BMI was a statistically significant moderator of MetS prevalence (*p* < 0.05). Abbreviations: BMI, body mass index (kg/m^2^); MetS, metabolic syndrome; OSA, obstructive sleep apnoea.
